# Genetic Control of Resistance to *Colletotrichum kahawae* in Coffee: Evidence of Polygenic Inheritance and Differential Host Genotype Responses to Pathogen Isolates

**DOI:** 10.3390/plants15132002

**Published:** 2026-06-28

**Authors:** Julio Quiroga-Cardona, Andreia Loureiro, Vítor Manuel Pinto Várzea, Claudia Patricia Flórez-Ramos, Maria do Céu Silva

**Affiliations:** 1 Plant Breeding, National Coffee Research Center, Cenicafé, Manizales 170009, Colombia; claudia.florez@cafedecolombia.com; 2 Faculty of Agricultural Sciences, University of Caldas, Manizales 170001, Colombia; 3 Centro de Investigação das Ferrugens do Cafeeiro, Instituto Superior de Agronomia, University of Lisboa, 1300-011 Lisboa, Portugal; andreialoureiro@isa.ulisboa.pt (A.L.); vitorvarzea@isa.ulisboa.pt (V.M.P.V.); 4 LEAF—Linking Landscape, Environment, Agriculture and Food and Associate Laboratory TERRA, Instituto Superior de Agronomia, University of Lisboa, 1349-017 Lisboa, Portugal

**Keywords:** *Coffea arabica*, *Colletotrichum kahawae*, coffee berry disease, polygenic inheritance, host resistance, plant breeding, pathotypes, Rume Sudan

## Abstract

*Colletotrichum kahawae* is the causal agent of coffee berry disease (CBD), a disease restricted to African countries producing *C. arabica*, which affects coffee production and is a potential phytosanitary threat for coffee plants growing at other latitudes. The inheritance of resistance to *C. kahawae* conferred by the *R* gene (*Ck-2/Ck-3*) in the Rume Sudan variety of coffee was previously reported as simple Mendelian (3:1). However, our results derived from an $F_{2}$ population of 10,180 hypocotyls, similar to the resistance observed in Rume Sudan against *C. kahawae*, reveals a polygenic involvement. Therefore, resistance to CBD cannot be explained by a Mendelian segregation model, since the observed phenotypic interactions reveals that resistance involves a system of genes [quantitative trait loci (QTLs)]. Similarly, the genetic potential for resistance of a wild genotype of *C. arabica* ET.56 to *C. kahawae* was evaluated, and its segregation was also evident in its phenotypic interactions, similar to that of Rume Sudan. However, differences in the phenotypic expression of their segregated populations potentially suggest that the genetic mechanisms responsible for resistance to *C. kahawae* in these two varieties are likely different. Finally, statistical analysis revealed the existence of the *C. arabica*/*C. kahawae* interaction and the consequent existence of pathotypes, demonstrating that resistance is not transversal but specific to *C. kahawae* isolates. This behavior is consistent with that previously reported based on the genetic diversity of *C. kahawae* and can be used in genetic improvement programs for *C. arabica* to enhance the development of varieties resistant to the disease.

## 1. Introduction

*Colletotrichum kahawae* Waller and Bridge [1] is a hemibiotrophic fungus [2,3] that is restricted to the African continent and is the causal agent of coffee berry disease (CBD) [4,5,6]. CBD affects the production of *C. arabica*, causing economic losses that can exceed 80% in susceptible varieties when climatic conditions favor the development of the fungus and when no preventive measures are taken to control the disease [7,8].

CBD epidemics are characterized by temperatures ranging between 15 °C and 25 °C and an air moisture content close to the saturation point (>95%) [9,10,11,12]. Although *C. kahawae* is not found in America, it is a potential threat to the production of coffee in this region because of the climatic conditions present in coffee-growing areas, which can favor the establishment and development of the fungus in the event of its eventual arrival. Coupled with this, varieties resistant to *C. kahawae* are not currently grown in countries producing *C. arabica* [6]. During epidemics, genetic resistance to *C. kahawae* can be durable, as documented for the Ruiru 11 and Batian varieties of *C. arabica* [13,14]. These varieties have been developed from diverse genetic sources, including the Timor Hybrid (TH), an interspecific hybrid between *C. arabica* and *C. canephora* [15,16] and Rume Sudan, a genotype of *C. arabica* derived from a wild population that has high phenotypic variability [17,18,19]. These two varieties (TH and Rume Sudan) have been reported to carry genes imparting resistance to *C. kahawae*, i.e., the *T* gene (*Ck-1*) in TH CIFC 1343 and the *R* gene (*Ck-2* and *Ck-3*) in Rume Sudan. Genetic studies on the resistance in these varieties have indicated that the segregation of the two genes is of the Mendelian type (3:1) exhibiting dominance [19,20,21]. However, the complexity of the disease, the difficulty in identifying differential genotypes and the occurrence of *plant-pathogen* interactions have prevented the establishment of a consensus in the scientific community regarding the nature of genetic resistance and its hereditary pattern [19,20,22,23,24].

In Colombia, researchers, as part of the Cenicafé Improvement Program, have been working for more than 30 years on the development of varieties with genetic resistance to *C. kahawae* from various sources [6,25], with TH CIFC 1343 as the main source [6,26]. Resistance to *C. kahawae* derived from TH CIFC 1343 was identified in Kenya in the 1970s [27,28]. Broad characterizations of disease resistance in genotypes derived from TH CIFC 1343 and other varieties of *C. arabica* have been carried out in Portugal at the CIFC (Centro de Investigação das Ferrugens do Cafeeiro in Portuguese) [6,29]. These characterizations were performed under controlled conditions via the hypocotyl inoculation method [30]. This method supports the identification of resistance to *C. kahawae* in nonclonal genotypes and is correlated (>0.73) with CBD resistance during epidemics [31]. Protecting cultivated Colombian coffee plants from potential diseases such as CBD through the development of varieties with genetic resistance has been a priority for Cenicafé. In Colombia, the cultivation of *C. arabica* plays an important role from economic, cultural and social perspectives for 560,000 families [32]. In the country, 830,000 hectares of land are used for growing coffee, and 88% of coffee varieties grown in the region are resistant to coffee rust (*Hemileia vastatrix* Berk and Broome) [33] and to different isolates of *C. kahawae*. These isolates are diverse in terms of their aggressiveness and geographic origin [6]. However, although the commercial varieties developed by Cenicafé currently grown in the Colombian coffee park are resistant to *C. kahawae*, this resistance originates from a single source—TH CIFC 1343.

Identifying new genetic sources of resistance to *C. kahawae* in *C. arabica* and the type of inheritance that governs it would contribute to more efficient selection of genotypes in early stages of evaluation. This is essential for the development of new varieties and to maintain resistance in coffee crops to CBD over time. Therefore, the objective of the present investigation was to identify the potential of new sources of resistance to *C. kahawae*, the corresponding genetic parameters and the segregation of resistance in different $F_{2}$ populations. The parameters were obtained from the evaluation of the resistance of two wild varieties, Rume Sudan (CCC81) and ET.56 (CCC1147) (Ethiopian wild *C. arabica*), from the Colombian Coffee Collection (CCC) to three isolates of fungus, which were diverse in terms of aggressiveness and geographical origin.

## 2. Materials and Methods

### 2.1. Location

The evaluated varieties were developed and established at the Naranjal Experimental Station (04°58′ N, 75°39′ W), located in the municipality of Chinchiná (Caldas-Colombia), at 1381 m above sea level (masl), with an average temperature of 21.4 °C, precipitation of 2782 mm per year and an average relative humidity of 77.5%. Evaluations of resistance to *C. kahawae* were carried out at the CIFC in Portugal in 2025.

### 2.2. Genotypes Evaluated

Thirty-three segregating populations ($F_{2}$) derived from three $F_{1}$ hybrids and their respective progenitors were evaluated (Table 1). The populations were obtained from crosses between susceptible varieties (Iapar-59 and Catuaí) and wild genotypes of *C. arabica*, Rume Sudan and ET.56, both of which are resistant to *C. kahawae*. The Caturra and SL.28 varieties were used as susceptibility control genotypes.

### 2.3. Isolates of C. kahawae

Three isolates of *C. kahawae*, previously characterized as diverse in terms of aggressiveness and geographic origin, were used [34,35,36]. These isolates belong to the biological collection of the CIFC (Table 2).

### 2.4. Inoculation with C. kahawae Isolates

Strength evaluations were performed using a methodology developed previously [31,38] and modified by the CIFC. For this purpose, hypocotyls 3 to 6 cm long (5–6 weeks of development) were selected and placed on nylon sponges moistened with water in plastic boxes, which acted as a humid chamber. The hypocotyls were subsequently inoculated with a suspension of *C. kahawae* conidia at a concentration of $2\times 10^{6}$ conidia per milliliter of water, which was quantified by a Neubauer chamber. The conidia were obtained from in vitro cultures of *C. kahawae* in malt extract agar medium (MEA) (Oxoid, 3.4%) and cultured for eight days at 22 °C. The conidia were subsequently suspended in sterile distilled water. The suspension was filtered twice through layers of sterile muslin cloth to eliminate the mycelium and then sprayed on each hypocotyl with an atomizer coupled to an air pressure pump (Pump type VDE 0530).

The inoculated hypocotyls were kept in a humid chamber for 48 h in complete darkness, after which a second inoculation was performed under the same conditions. During the first four days of incubation, the humidity of the chambers was maintained constant, with an average temperature of 22 °C; following this period, the temperature was maintained at 19 °C, the relative humidity was maintained close to the saturation point, and the photoperiod was 12 h. The infection process was monitored for four weeks, during which time the hypocotyls were classified according to the severity scale [38,39] modified by the CIFC [6]. Each hypocotyl infected with *C. kahawae* was classified as Resistant if it developed Class 1, 2 or 3 infection or as Susceptible if it developed Class 4 (necrotrophic phase) infection. Class 1: Development of small greenish lesions up to 1–2 narrow brown lesions and lesions up to 0.5 mm wide. Class 2: Development of brown lesions that exceed 0.5 mm. Lesions coalesce. The formation of black lesions is not frequent; however, they can occur. Class 3: Large brown lesions with numerous black spots and/or black lesions. Black lesions can completely surround the stem without death of the upper part. Class 4: Black lesions completely surround the stem. The upper part of the hypocotyl dies.

Resistance was quantified based on the cumulative percentage of hypocotyls that developed Class 4 infections (necrotrophic phase) between nine and 28 days after inoculation (DAI).

### 2.5. Evaluation of Resistance to C. kahawae and Genetic Parameters

From the quantification of the percentage of resistant (Class 1 + Class 2 + Class 3) and susceptible hypocotyls (Class 4), the following parameters were estimated:

(a) The progression curve of the disease of each population was categorized according to the classification proposed in a previous study [6] (Table 3).

(b) From the observed phenotypic resistance patterns, the pattern of inheritance of resistance to *C. kahawae* was estimated. For this purpose, a chi-square ($\chi^{2}$) goodness-of-fit test was performed to compare the obtained phenotypic frequencies (Class 1 + Class 2 + Class 3-resistant hypocotyls vs. Class 4-susceptible hypocotyls) with the expected theoretical phenotypic proportions according to Mendelian segregation.

(c) For each combination (genotype–isolate), the genetic parameters of resistance to *C. kahawae* were estimated through a linear mixed effects model (LMM) using the lme4 package in R v1.1-14. In the model, the response variable corresponded to the percentage of hypocotyls resistant to *C. kahawae* (Class 1 + Class 2 + Class 3). The genotype (CCC), *C. kahawae* isolation and the genotype–isolate interaction were considered random effects.

From the model, the variance components were estimated: (a) variance attributed to the genetic component $\left(\sigma_{G}^{2}\right)$, (b) variance attributed to isolation $\left(\sigma_{I}^{2}\right)$, (c) variance in the interaction between the genotype and isolate of *C. kahawae* $\sigma_{G}^{2}$ × $\sigma_{I}^{2}$ and (d) residual variance $\left(\sigma_{e}^{2}\right)$. The values obtained were derived from the quantification of the relative weight of each component of variance in the genetic model and were used to theoretically explain the resistance to each of the isolates of *C. kahawae* (Ang29, Cam1, and Que2).

$$\mathrm{Genetic model:}\;\sigma_{F}^{2}=\sigma_{G}^{2}+\sigma_{I}^{2}+\sigma_{G\times I}^{2}+\sigma_{e}^{2}$$

$\sigma_{F}^{2}$: Total phenotypic variation observed in the expression of resistance to *C. kahawae* among $F_{2}$ populations. $\sigma_{G}^{2}$: Variation in the expression of resistance to *C. kahawae* that is exclusively due to genetic differences between $F_{2}$ populations. $\sigma_{I}^{2}$: Variation in resistance due to differences in virulence or aggressiveness between *C. kahawae* isolates (Ang29, Cam1, and Que2). $\sigma_{G\times I}^{2}$: Variation due to the expression of resistance to *C. kahawae* influenced by the interactions between populations and isolates. $\sigma_{e}^{2}$: Variation not explained by the above factors, which include experimental error and uncontrolled factors within the experiment.

(d) From the estimated variance components, broad-sense heritability ($H^{2}$) was calculated as the proportion of the total phenotypic variance ($\sigma_{F}^{2}$) attributable to the genetic effects of resistance to *C. kahawae* ($H^{2}=\frac{\sigma_{G}^{2}}{\sigma_{F}^{2}}$) and the respective confidence intervals (CIs), with 95% accuracy from 1000 bootstrapping simulations.

## 3. Results

### 3.1. Classification of Hypocotyls by Classes of Resistance to C. kahawae

**Figure 1:**
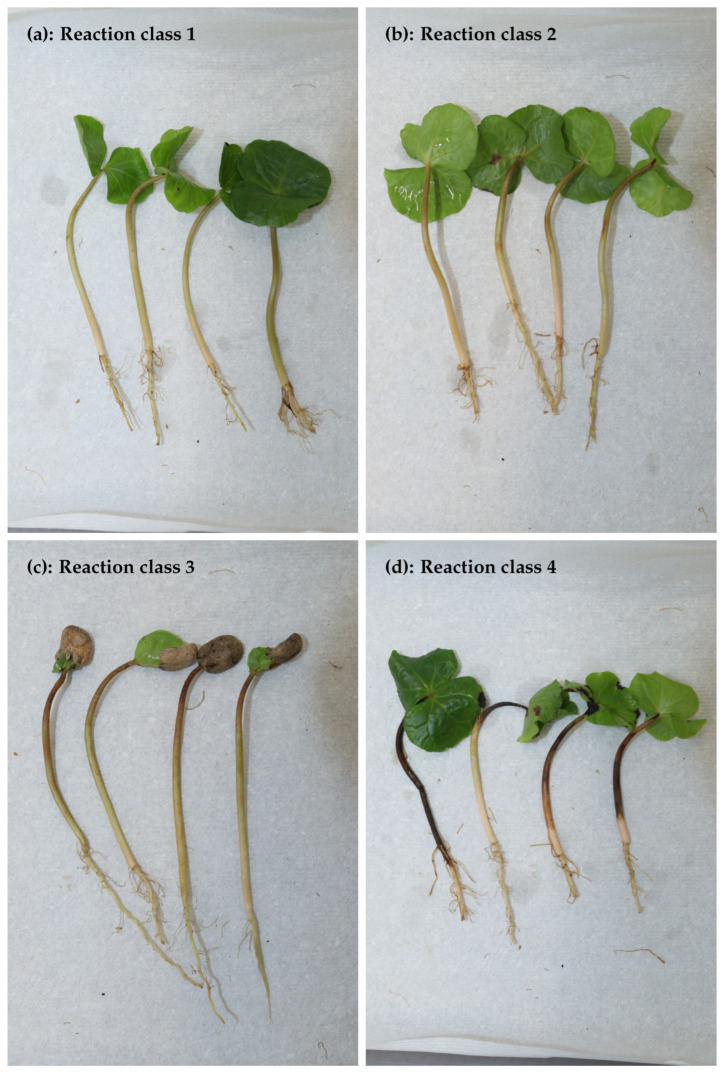
Reaction classes of *C. arabica* genotypes, 28 days post-inoculation with *C. kahawae* isolates, evaluated using the van der Graaff scale [39]. (**a**) Reaction class 1, development of small narrow brown lesions up to 0.5 mm wide. (**b**) Reaction class 2, development of brown lesions that exceed 0.5 mm, along with coalescence of lesions (**c**) Reaction class 3, large brown coalescent lesions (**d**) Reaction class 4, black lesions that surround the stem (necrotrophic phase). Death of the upper part of the hypocotyl.

Phenotypic reactions of infection were clustered in each hypocotyl 28 DAI. Reactions were classified as resistant in class 1, 2, or 3, or as susceptible if a class 4 infection (necrotrophic phase) developed. Reaction class types, based on the van der Graaff scale, were observed in the coffee genotypes (Figure 1).

### 3.2. Resistance to C. kahawae in Progenitors of the $F_{2}$ Population

**Figure 2:**
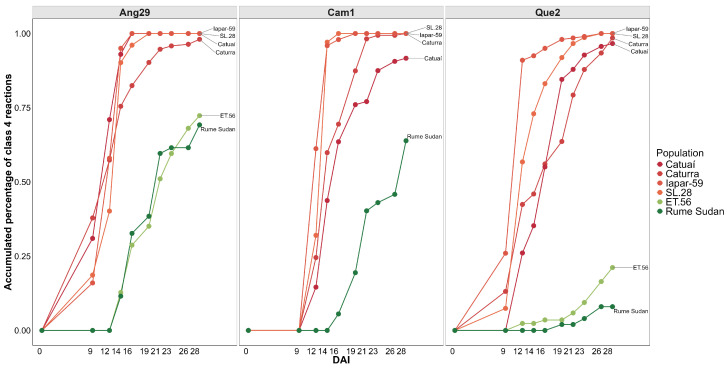
Cumulative percentage of progenitors and control genotypes exhibiting susceptibility (Class 4) to the three isolates of *C. kahawae*. Ang29: Cumulative percentage of hypocotyls with susceptibility (Class 4) to the isolate of Angola origin. Cam1: Cumulative percentage of hypocotyls with susceptibility (Class 4) to the isolate of Cameroon origin. Que2: Cumulative percentage of hypocotyls with susceptibility (Class 4) to the isolate from Kenya.

The progenitors of the evaluated populations developed contrasting resistance patterns to the three *C. kahawae* isolates used (Figure 2). Notably, 30.8% and 27.7% of hypocotyls of Rume Sudan and ET.56, respectively, were resistant to the *C. kahawae* isolate of Angola origin (Ang29), an isolate considered highly aggressive. On the basis of these results, the two varieties were classified in the category characterized by low resistance to *C. kahawae* (Ang29) (Figure 2).

The resistance of Rume Sudan to the isolate from Cameroon (Cam1) was low, with 36.1% hypocotyls exhibiting resistance. In this study, it was not possible to evaluate the resistance of ET.56 to the Cam1 isolate because the number of seeds that germinated was insufficient for the data to be statistically reliable. However, in previous studies conducted by Cenicafé, ET.56 was reported to be highly resistant to *C. kahawae* [6]. On the basis of these reports, the ET.56 variety was used as a potential source of resistance to *C. kahawae*.

On the other hand, the resistance of Rume Sudan and ET.56 varieties to the isolate of *C. kahawae* of Kenyan origin (Que2) was 92.0% and 78.8%, respectively. For this isolate, both genotypes were classified in the category of highest resistance (Figure 2). The progenitors susceptible to *C. kahawae* and the control genotypes (Caturra and SL.28 varieties) were classified in the ‘Susceptibility’ category, with necrotrophic reactions (>92%) to the three isolates evaluated (Ang29, Cam1 and Que2).

The pattern of resistance to *C. kahawae* during the first 14 DAI differed between the resistant varieties (Rume Sudan, ET.56, Iapar-59, and Catuaí) and the control varieties (Caturra and SL.28) in terms of the number of hypocotyls resistant to different *C. kahawae* isolates. During this initial period, the hypocotyls of the wild varieties, Rume Sudan and ET.56, did not experience necrotrophic reactions (i.e., were not susceptible). In contrast, the Caturra, Catuaí, Iapar-59 and SL.28 genotypes developed early and accelerated susceptibility (Class 4) to *C. kahawae* (Figure 2).

### 3.3. Segregation of Resistance to C. kahawae in $F_{2}$ Populations

#### 3.3.1. Segregation of Resistance of the Iapar-59 × Rume Sudan Population to the Ang29 Isolate

**Figure 3:**
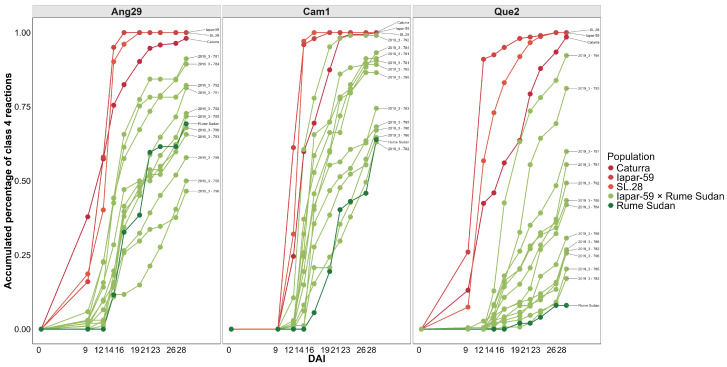
Cumulative percentage of hypocotyls with susceptibility (Class 4) among segregating genotypes $F_{2}$ of Iapar-59 × Rume Sudan vs. progenitors resistant and susceptible to three isolates of *C. kahawae*. Ang29: Cumulative percentage of hypocotyls with susceptibility (Class 4) to the isolate of Angola origin. Cam1: Cumulative percentage of hypocotyls with susceptibility (Class 4) to the isolate of Cameroon origin. Que2: Cumulative percentage of hypocotyls with susceptibility (Class 4) to the isolate from Kenya. DAI: Days after inoculation.

A total of 1114 $F_{2}$ hypocotyls were evaluated for resistance to the isolate of Angola origin; the hypocotyls were obtained from eleven $F_{1}$ hybrid plants. Resistance in this population exhibited high variability similar to that expected in a segregating population. The percentage of resistant hypocotyls ranged between 8.8% and 53.9%. Thus, the hybrids originated from populations with moderate and very low resistance to the Ang29 isolate. Forty-five percent of the hybrid plants (2019_3 – 783, 786, 788, 795 and 796) segregated into populations with higher resistance compared with the resistant progenitor variety, Rume Sudan (Figure 3).

#### 3.3.2. Segregation of Resistance of the Iapar-59 × Rume Sudan Population to the Cam1 Isolate

A total of 1011 $F_{2}$ hypocotyls from eleven $F_{1}$ hybrids were evaluated for resistance to the isolate of Cameroon origin, and the percentages of hypocotyls resistant to *C. kahawae* were distributed in three groups (Figure 3). Each group presented resistance patterns distributed between those of the resistant progenitor (Rume Sudan) and the susceptible progenitor (Iapar-59) varieties. The first group comprised of the $F_{1}$ 2019_3 – 792 plants and completely segregated into susceptible hypocotyls, which also included the progenitor Iapar-59 variety.

The second group comprised the segregating hypocotyls from the hybrid plants 2019_3 – 781, 784, 785, 788 and 791. The percentages of resistant hypocotyls in this group ranged between 6.8% and 13.5%. The third group comprised the segregating hypocotyls derived from the $F_{1}$ 2019_3 – 782, 783, 786, 795 and 796 plants. The percentages of resistant segregating hypocotyls in this group were between 25.5% and 36.9%, and the resistance patterns were similar to those of the resistant progenitor, Rume Sudan (Figure 3).

#### 3.3.3. Segregation of Resistance of the Iapar-59 × Rume Sudan Population to the Que2 Isolate

A total of 2559 segregating hypocotyls obtained from thirteen hybrid plants were evaluated for resistance to the Kenyan isolate. On the basis of the percentages of hypocotyls resistant to the Que2 isolate, the plants were divided into three groups. The first group comprised segregants derived from the $F_{1}$ 2019_3 – 793 and 794 plants with percentages of resistant hypocotyls close to those for the susceptible progenitor variety (Iapar-59). The second group comprised segregating populations derived from the hybrid plants, 2019_3 – 781, 784, 791, 792 and 795. These populations exhibited resistance percentages that ranged between 40.1% and 58%. The third group comprised those obtained from the hybrids, 2019_3 – 782, 783, 785, 786, 788 and 796. In these populations, the percentage of resistant hypocotyls was higher than 69% (Figure 3).

#### 3.3.4. Segregation of Resistance of the Rume Sudan × Catuaí Population to the Ang29 Isolate

**Figure 4:**
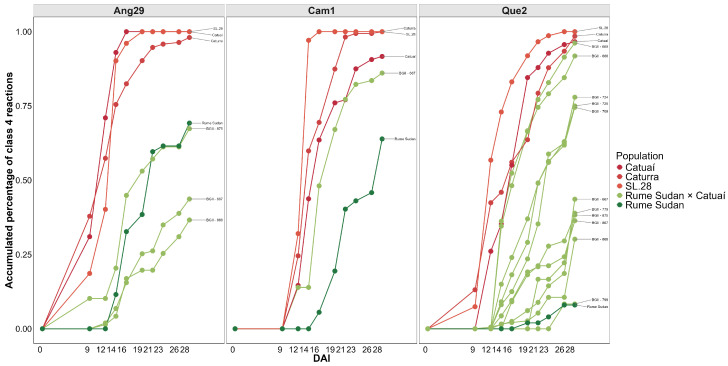
Cumulative percentage of hypocotyls with susceptibility (Class 4) among segregating genotypes $F_{2}$ of Rume Sudan × Catuaí vs. progenitors resistant and susceptible to three isolates of *C. kahawae*. Ang29: Cumulative percentage of hypocotyls with susceptibility (Class 4) to the isolate of Angola origin. Cam1: Cumulative percentage of hypocotyls with susceptibility (Class 4) to the isolate of Cameroon origin. Que2: Cumulative percentage of hypocotyls with susceptibility (Class 4) to the isolate from Kenya. DAI: Days after inoculation.

A total of 223 hypocotyls from this population were evaluated. The population obtained from the $F_{1}$ BGII – 875 plants contained 32.7% resistant hypocotyls, a percentage equal to that obtained for the resistant parent (Rume Sudan). The populations obtained from the BGII – 667 and 868 hybrids exhibited higher percentages of resistance than the resistant parent variety, similar to that observed in plants from the Iapar-59 × Rume Sudan population when they were evaluated for resistance to the same isolate (Figure 4).

#### 3.3.5. Segregation of Resistance of the Rume Sudan × Catuaí Population to the Cam1 Isolate

A total of 79 segregating hypocotyls derived from a single hybrid, BGII – 667, were evaluated. The percentage of resistant hypocotyls in this population was 13.9%, indicating intermediate resistance among the progenitors (resistant and susceptible to *C. kahawae*) (Figure 4).

#### 3.3.6. Segregation of Resistance of the Rume Sudan × Catuaí Population to the Que2 Isolate

A total of 1321 segregating hypocotyls were evaluated and, based on their resistance to *C. kahawae* (Que2), were divided into four groups. The first group comprised segregants obtained from the BGII – 668 and 669 hybrids that were classified as susceptible (Class 4). The second group comprised segregants whose percentage of resistant hypocotyls was between 22.1% and 25.5%. These hypocotyls were derived from the BGII – 709, 724 and 725 hybrids, with resistance patterns similar to that of the resistant progenitor, Rume Sudan; these segregants were derived from the hybrid BGII – 799 (Figure 4).

#### 3.3.7. Segregation of Resistance of the Iapar-59 × ET.56 Population to the Ang29 Isolate

The resistance pattern of the hypocotyls of this segregating population was similar to that observed for the populations derived from Iapar-59 × Rume Sudan. Segregation of resistance to Ang29 was observed in 983 $F_{2}$ hypocotyls of the Iapar-59 × ET.56 population. Compared with the susceptible parent variety (Iapar-59), the hybrids 2019_3 – 745, 746, 752 and 755 segregated into populations whose phenotypic resistance was higher than that of the susceptible parent variety (Iapar-59) but lower than that of the resistant wild genotype (ET.56).

**Figure 5:**
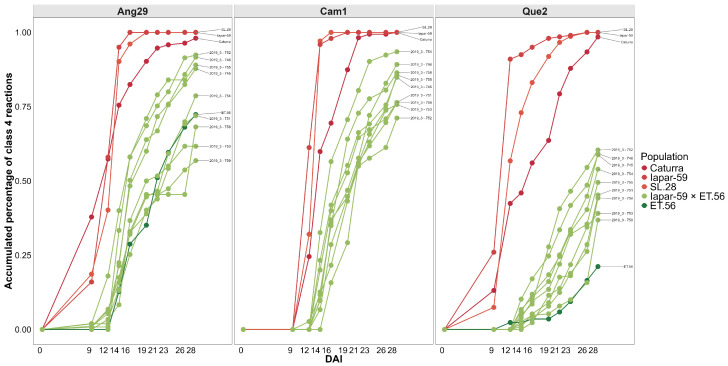
Cumulative percentage of hypocotyls with susceptibility (Class 4) among the segregating genotypes $F_{2}$ of Iapar-59 × ET.56 vs. progenitors resistant and susceptible to three isolates of *C. kahawae*. Ang29: Cumulative percentage of hypocotyls with susceptibility (Class 4) to the isolate of Angola origin. Cam1: Cumulative percentage of hypocotyls with susceptibility (Class 4) to the isolate of Cameroon origin. Que2: Cumulative percentage of hypocotyls with susceptibility (Class 4) to the isolate of Kenyan origin. DAI: Days after inoculation.

The populations derived from the 2019_3 – 754 and 751 hybrids expressed resistance percentages similar to those of the ET.56 progenitor, and the populations represented by the hybrids 2019_3 – 753, 758 and 759 expressed transgressive segregation similar to that of the resistant progenitor (Figure 5). This pattern was common in the three hybrid populations when evaluated for resistance to the Ang29 isolate (Figure 3, Figure 4 and Figure 5).

#### 3.3.8. Segregation of Resistance of the Iapar-59 × ET.56 Population to the Cam1 Isolate

The phenotypic resistance of 996 $F_{2}$ hypocotyls derived from nine hybrids to the Cam1 isolate was evaluated. The percentages of resistant hypocotyls were between 6.5% and 28.8%, and these values were higher than those obtained for the susceptible progenitor variety, Iapar-59 (Figure 5).

#### 3.3.9. Segregation of Resistance of the Iapar-59 × ET.56 Population to the Que2 Isolate

The percentages of hypocotyls resistant to the Que2 isolate among the 1894 segregating hypocotyls ranged between 39.6% and 63.2%. The resistance of all $F_{2}$ populations of Iapar-59 × ET.56 was categorized as intermediate resistance and differed from that observed in the other populations (Iapar-59 × Rume Sudan and Rume Sudan × Catuaí), and the percentages of resistant hypocotyls were scattered and less concentrated (Figure 5). The effects of resistance mechanisms other than those of Rume Sudan could not be ruled out.

### 3.4. Statistical Analysis

Analysis of variance of the percentage of resistant hypocotyls of each $F_{2}$ population of the sources of resistance to *C. kahawae* derived from each hybrid plant from each $F_{1}$ group (Iapar-59 × Rume Sudan, Rume Sudan × Catuaí and Iapar-59 × ET.56) was performed. $F_{1}$ hybrids were segregated into populations with different levels of resistance even when derived from the same progenitors. The *C. kahawae* isolates used (Ang29, Cam1 and Que2) differed in aggressiveness, and significant differences were detected regarding the interaction between the $F_{2}$ population and the G × I (Genotype × Isolates) interactions. The percentage of resistant hypocotyls differed significantly according to the isolate of *C. kahawae* used in the inoculation (Table 4).

In the absence of documented information on possible maternal effects, the analysis of variance revealed that resistance to *C. kahawae* is nuclear and not cytoplasmic. Therefore, when Rume Sudan is used as a female or male parent in a cross with a susceptible plant variety, it is assumed that the final response is the same.

The significant (p<0.05) differences detected between the $F_{2}$ populations suggest that although the segregating populations originate from the same source of resistance and a susceptible progenitor variety, different resistance patterns are expressed. Therefore, to determine the segregating pattern of resistance to each *C. kahawae* isolate (Ang29, Cam1 and Que2), each $F_{2}$ population and its observed resistance percentage were analyzed separately. For this analysis, a total of 10,180 segregating hypocotyls were used (Iapar-59 × Rume Sudan: n = 4684; Rume Sudan × Catuaí: n = 1623; Iapar-59 × ET.56: n = 3873) (Table 1) and the phenotypic frequencies of resistance observed in each population were compared with the expected theoretical Mendelian proportions through a chi-square test ($\chi^{2}$).

### 3.5. Exploration of Possible Types of Segregation of Resistance to C. kahawae

#### 3.5.1. Segregation of Resistance to the Ang29 Isolate

In 96% of the evaluated $F_{2}$ populations, the observed phenotypic frequencies did not fit the expected 3:1 theoretical ratio, indicating that resistance to the Ang29 isolate is not governed by complete monogenic dominance (p<0.05) (Table 5 and Table S1). Instead, alternative genetic models were identified across different progenies.

A monogenic recessive inheritance model (1:3 ratio) was confirmed in specific populations, where the observed frequencies aligned statistically with theoretical expectations (p>0.05). This behavior was evident in 5 out of 11 populations from Iapar-59 × Rume Sudan, 3 out of 9 from Iapar-59 × ET.56, and 1 out of 3 from Rume Sudan × Catuaí (Table 5 and Table S2). Furthermore, a complementary epistatic model (9:7 ratio) was validated (p>0.05) in 2 out of 11 populations of Iapar-59 × Rume Sudan, 1 out of 9 of Iapar-59 × ET.56, and 2 out of 3 of Rume Sudan × Catuaí (Table 5 and Table S3). Lastly, a duplicate dominant epistatic interaction (15:1 ratio) was significantly supported (p>0.05) in some progenies, specifically in 2 out of 11 populations from Iapar-59 × Rume Sudan and 2 out of 9 from Iapar-59 × ET.56, whereas it was entirely absent in the Rume Sudan × Catuaí cross (Table 5 and Table S4).

#### 3.5.2. Segregation of Resistance to the Cam1 Isolate

Consistent with the trends observed for Ang29, none of the segregating populations conformed to the monogenic complete dominant model (3:1 ratio), confirming significant deviations from this type of inheritance (p<0.05) (Table 5 and Table S5).

The monogenic recessive model (1:3 ratio) was successfully validated (p>0.05) in 4 out of 11 populations from Iapar-59 × Rume Sudan and 4 out of 9 from Iapar-59 × ET.56, while the single evaluated population from Rume Sudan × Catuaí failed to fit this model (Table 5 and Table S6). Regarding dihybrid models, the complementary epistasis model (9:7 ratio) was statistically rejected across all evaluated populations (p<0.05), showing no alignment with theoretical expectations (Table 5 and Table S7). Conversely, the duplicate dominant model (15:1 ratio) was significantly supported (p>0.05) in 4 out of 11 populations from Iapar-59 × Rume Sudan and 1 out of 9 from Iapar-59 × ET.56, but was not detected in the Rume Sudan × Catuaí progeny (Table 5 and Table S8).

#### 3.5.3. Segregation of Resistance to the Que2 Isolate

In contrast to the other isolates, a monogenic complete dominant pattern (3:1 ratio) was successfully validated (p>0.05) in certain progenies, including 5 out of 13 populations from Iapar-59 × Rume Sudan and 1 out of 9 from Rume Sudan × Catuaí, whereas 100% of the populations from Iapar-59 × ET.56 rejected this model (Table 5 and Table S9).

The monogenic recessive model (1:3 ratio) was verified (p>0.05) in only three specific populations: 1 out of 13 from Iapar-59 × Rume Sudan and 3 out of 9 from Rume Sudan × Catuaí (Table 5 and Table S10). On the other hand, the complementary epistatic model (9:7 ratio) proved to be a prevalent pattern for this isolate, showing a significant fit (p>0.05) in 3 out of 13 populations from Iapar-59 × Rume Sudan, 5 out of 9 from Iapar-59 × ET.56, and 3 out of 9 from Rume Sudan × Catuaí (Table 5 and Table S11). Finally, the duplicate dominant gene action model (15:1 ratio) was statistically supported (p>0.05) in a few remaining populations, notably in 1 out of 13 from Iapar-59 × Rume Sudan and 2 out of 9 from Rume Sudan × Catuaí (Table 5 and Table S12).

In conclusion, the fact that none of the tested Mendelian segregation models (3:1, 1:3, 9:7, or 15:1) consistently fit all the evaluated $F_{2}$ populations and their respective segregants across the three *C. kahawae* isolates highlights a complex genetic architecture. This lack of a uniform monogenic or simple dihybrid pattern across different genetic backgrounds indicates that resistance is not governed by a single major locus or simple epistatic pairs. Instead, these findings collectively demonstrate that resistance to these isolates is a polygenic trait, likely controlled by multiple quantitative trait loci (QTLs) or a complex network of minor genes whose phenotypic expression and segregation ratios vary depending on the specific parental cross and the fungal isolate involved.

### 3.6. Genetic Parameters of Resistance to C. kahawae

#### 3.6.1. Classification of Isolates

The isolates were grouped according to the aggressiveness observed in the evaluated populations on the basis of the source of resistance used (Rume Sudan and ET.56). The statistical criterion used for this grouping was the minimum significant difference (p<0.05) obtained from the corresponding analysis of variance (Table 4). For each source of resistance, based on the aggressiveness, the Ang29, Cam1 and Que2 isolates were classified into two categories with statistical differences (Table 6).

#### 3.6.2. Linear Mixed Models (Random Effects)

The analysis of variance (Table 4) was complemented with the use of linear mixed models (random effects). For this, two models were adjusted: The first model was adjusted based on the available information and without considering the interaction between the factors (Genotype × Isolate). The second model was adjusted considering the interaction.

Model comparison indicated that including the Genotype × Isolate interaction significantly improved model fit in all populations (ΔAIC=2.4–9.1; p<0.05), showing clear statistical superiority over the simpler model. This result revealed that interactions between the two resistance sources (Rume Sudan and ET.56) and the evaluated isolates (Ang29, Cam1, and Que2) significantly contribute to variations in phenotypic resistance to *C. kahawae* among hypocotyls. This firmly indicates that the resistance responses in these populations were isolate-specific (Table S13).

#### 3.6.3. Deduction of the Genetic Parameters Related to the Resistance of *C. arabica* to *C. kahawae*

Based on the statistical results of the interaction model, the total variability observed in the percentage of hypocotyls expressing resistance to *C. kahawae* was deduced.

The analysis of variance components revealed that genetic variation had the strongest influence on the resistance to *C. kahawae* in the three populations evaluated. In the genetic model of the Rume Sudan × Catuaí population, genetic variation exhibited the highest contribution, accounting for 64.2% of the total variance ($H^{2}=0.642$).

Additionally, the Iapar-59 × Rume Sudan population exhibited a heritability magnitude of $H^{2}=0.423$. This difference in the magnitude of the mean heritability is probably related to cytoplasmic effects involved in the expression of resistance to *C. kahawae*. However, the corresponding CIs indicate that this is only a preliminary approximation; therefore, it must be validated.

In terms of the interactions that occurred between the populations and the isolates, the interaction for the segregating populations derived from Rume Sudan × Catuaí exhibited the highest contribution; this component contributed to 25.1% of the total variance observed, which was also the highest compared with that of other populations to the isolates. It is likely that the resistance of the plants derived from this population is more specific than that of other populations to the *C. kahawae* isolates evaluated.

On the other hand, in the three populations, the uncontrolled variance ($\sigma_{e}^{2}$) was less than 12.6%. However, although all the hypocotyls had the same duration of vegetative development, it is highly likely that within each $F_{2}$ group of the Iapar-59 × ET.56 population, small physiological differences were present. These small differences that were not detected owing to the high degree of visual complexity contributed to 12.6% of the experimental error in this specific case. Moreover, the value for the experimental control of greater than 92.6% confirmed the reliability of the results obtained (Table 7 and Table S14).

### 3.7. Selection of the Best Genotypes Based on Their Resistance to C. kahawae

The individual differences in resistance to *C. kahawae* between each of the $F_{2}$ populations were calculated to identify the best $F_{1}$ hybrids. This is also known as the random effect or the deviation of the average resistance of a population with respect to the general average of the response variable. The results derived from the three hybrid populations and their segregating populations revealed that compared with the susceptible progenitors, the plants derived from the Rume Sudan and ET.56 varieties were genetically superior in terms of resistance to *C. kahawae*, and in some cases, the resistance was superior compared with that of the resistance donor source (Table S15).

The commercial varieties that were used as progenitors and susceptibility controls were consistently located at the lower end of the performance ranking, presenting negative values between −0.356 and −0.499, reflecting their indisputable susceptibility to all the isolates evaluated (Table S15). In contrast, the $F_{2}$ populations and the donor sources resistant to *C. kahawae* presented values that were close to zero and positive. These results validate the potential resistance of the Rume Sudan and ET.56 varieties against the three isolates of *C. kahawae*.

### 3.8. Effects of the Genotype × Isolate Interaction

**Figure 6:**
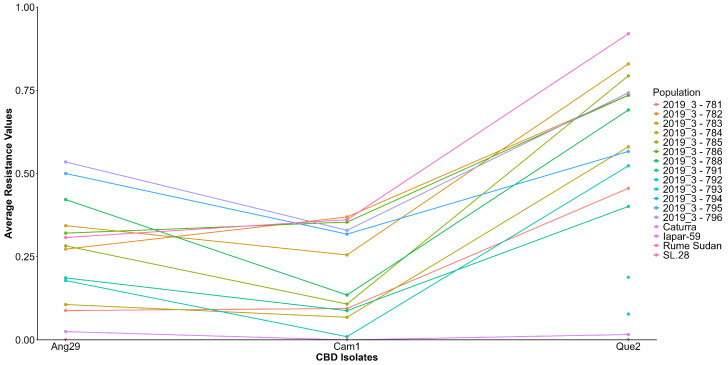
Graphical representation of the G × I interaction of the Iapar59 × Rume Rume population and the isolates Ang29, Cam1, and Que2. X-axis: Isolates of *C. kahawae*; Y-axis: Average resistance of *C. arabica* populations.

**Figure 7:**
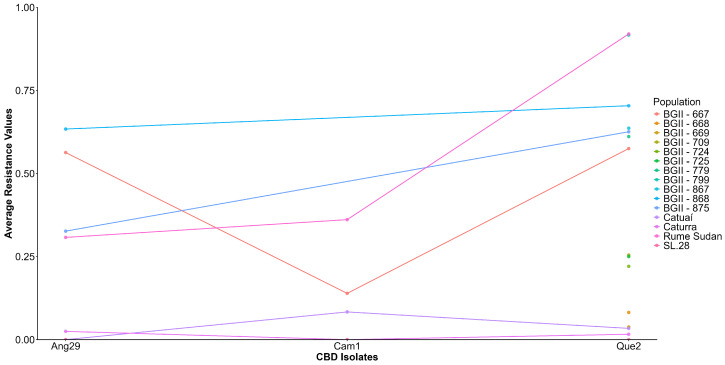
Graphical representation of the G × I interaction of the Rume Sudan × Catuaí population and the isolates Ang29, Cam1, and Que2. X-axis: Isolates of *C. kahawae*; Y-axis: Average resistance of *C. arabica* populations.

**Figure 8:**
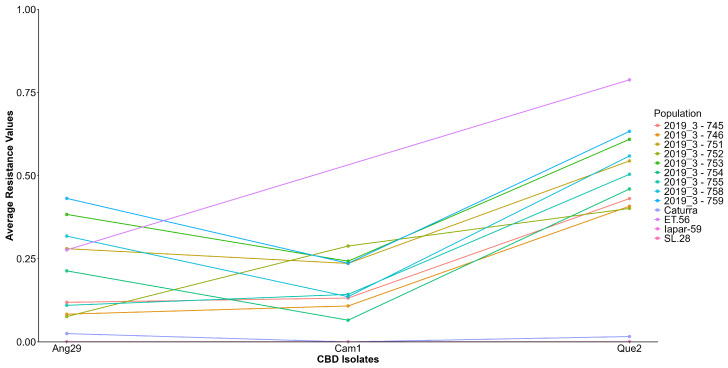
Graphical representation of the G × I interaction of the Iapar59 × ET.56 population and the isolates Ang29, Cam1, and Que2. X-axis: Isolates of *C. kahawae*; Y-axis: Average resistance of *C. arabica* populations.

In the three hybrid populations, as indicated by the respective analyses and the estimation of variances, a strong influence of the G × I interaction component was identified (Figure 6, Figure 7 and Figure 8). The interaction occurred in populations with some degree of resistance to *C. kahawae*, that is, the segregating populations and the resistant progenitor varieties, Rume Sudan and ET.56. Resistance to *C. kahawae* is not cross-sectional among isolates; in contrast, the pattern exhibits specificity. In the case of susceptible progenitors and controls, there was no interaction; that is, the genotypes exhibited the same resistance pattern (susceptibility) for the three isolates evaluated (Ang29, Cam1 and Que2).

## 4. Discussion

### 4.1. Sources of Genetic Resistance to C. kahawae

The TH CIFC 832/2 is resistant to some *C. kahawae* isolates (Cenicafé; Unpublished data), and this resistance is not present in all the populations derived from it. This has been demonstrated for the *C. arabica* variety, CIFC H361 (Villa Sarchi CIFC 971/10 × TH CIFC 832/2), specifically, the derivatives of plant number four (H361-4) that originated from the T5296 population (CATIE) and that were evaluated at the CIFC with the identifiers CIFC 16718, 16719, 16721 and 16722 [40]. In these cases, plants that were susceptible to *C. kahawae* isolates from Rwanda and Zimbabwe were identified. The Iapar-59 variety originated from the H361-4 plant [41]. Therefore, the susceptibility of Iapar-59 to *C. kahawae* is normal within the populations of *C. arabica* derived from the H361 hybrid. Similarly, it has been documented for TH CIFC 1343, which is resistant to some isolates of *C. kahawae*, but advanced progenies of this variety can exhibit susceptibility to other isolates [6,42].

For the Rume Sudan variety, the first characterization of resistance to *C. kahawae* revealed its potential as a source of CBD resistance genes. Rume Sudan plants exhibit high genetic variability [17]. Therefore, resistance to *C. kahawae* cannot be considered a universal parameter. A high percentage of fruits of the Rume Sudan plants do not develop CBD [18,19]. Similarly, the genetic improvement program of the Coffee Research Station in Lyamungu reported that some Rume Sudan plants are less affected by *C. kahawae* compared with some varieties of *C. arabica*, such as K7, Padang, Sidamo and some SL (Scott Laboratories) varieties [38,43,44,45,46,47].

The results obtained in the present investigation are consistent with those previously reported by studies performed under controlled conditions that, based on the average degree of infection of *C. kahawae*, the plants derived from the Rume Sudan variety can be classified as genotypes exhibiting resistance to the disease [20,31,43,48].

Resistance of plants derived from the ET.56 wild genotype of the Colombian Coffee Collection to *C. kahawae* has been detected during epidemics in Cameroon [49]. Although resistance of ET.56 to different isolates of *C. kahawae* from Cameroon, Kenya and Zimbabwe was previously evaluated under laboratory conditions via the hypocotyl test [6], this is the first detailed report of the pattern of resistance of ET.56 and its progeny to *C. kahawae*.

### 4.2. Segregation of Resistance to C. kahawae

At least three genes in *C. arabica* are involved in the expression of resistance to *C. kahawae*: The *T* gene or *Ck-1* in TH CIFC 1343 [20,21], the *k* gene in the K7 variety [20] and the *R* gene (*Ck-2* and *Ck-3*) in Rume Sudan and Pretoria [20,22]. The dominance and recessiveness of the genes and the number of genes involved in imparting resistance to *C. kahawae* have been debated [50].

A previous study [39] reported that inferring the genetic behavior of resistance to *C. kahawae* on the basis of observations made at very large laboratory scales (12 classes) is not adequate because the results obtained can be underestimated or overestimated. In addition, the conclusions reached in pioneering studies on resistance to *C. kahawae* could be questionable, as analyses were performed by measuring the extent of the disease using unadjusted scales [50]. Therefore, at very large scales, the populations for which measurements are made must be sufficiently large. Using large populations contributes to the reliability of the conclusions obtained in studies of segregating populations [51].

Studies confirming the dominance of the *R* gene were carried out in $F_{2}$ populations of *C. arabica* derived from the Rume Sudan variety. The results obtained were based on the use of the scale questioned previously [50] and a small population (segregants and progenitors) [22,48].

The results of the present investigation do not conform to those previously reported regarding the behavior of the *R* gene derived from Rume Sudan. The results were obtained with the adjusted scale proposed by [39] and based on observations made for $F_{2}$ populations derived from different $F_{1}$ hybrids (between 9 and 13 hybrids per $F_{1}$ cross). The hybrids acted as biological repetitions, and the segregation of each suggests that the segregation of the resistance of the *R* gene present in Rume Sudan identified by the CCC does not conform to the known Mendelian segregation pattern.

Analysis of variance (Table 4) revealed that each $F_{2}$ group was heterogeneous, with highly significant differences between them (p<0.05). Therefore, each segregating group should be considered independently and not as a single population. The low repeatability regarding the results of the expected theoretical segregation of each hybrid plant based on the chi-square ($\chi^{2}$) goodness-of-fit tests reveal that the resistance to *C. kahawae* derived from Rume Sudan is quantitative or polygenic. Therefore, its inheritance is more complex and goes beyond the theoretical Mendelian segregation pattern.

Genes with quantitative effects can have a cumulative effect on the expression of a trait; therefore, the greater the number of genes in a genotype, the greater the sum of their effects on the trait in question. This behavior has been detected in $F_{2}$ populations of wheat (*Triticum aestivum* L.), where genes that control the color of the endosperm exhibit complex segregation patterns [52,53]. *T. aestivum* plants carrying genes *R1 R1 R2 R2 R3 R3* × *r1 r1 r2 r2 r3 r3* segregate at a 63:1 ratio (63 grains of red wheat:1 grain of white wheat). In this case, the red color of the endosperm intensifies depending on the number of *R* genes that are present, which can range from one to six [52]. Therefore, multiple genes that influence a trait can express different degrees of dominance, which results in complex phenotypic variations [54].

The considerable variation in the observed segregation pattern of resistance to *C. kahawae* makes it impossible to fit a theoretical Mendelian segregation model. This variation suggests that resistance to *C. kahawae* is due to quantitative effects, possibly epistatic effects that result in polygenic inheritance. This type of resistance and its inheritance is based on a combination of loci with variable effects that contribute to phenotypes with different levels of resistance [55].

The epistatic effects (the effect or effects of a gene that depend on the presence of other genes) that may be present in the *C. arabica*/*C. kahawae* model could explain why the observed resistance does not conform to the Mendelian model and could be similar to populations exhibiting transgressive resistance. These populations exhibit resistance greater than or equal to that of their parents, possibly because of the combination of minor effect alleles that interact in a complex way, a phenomenon that has been observed in other *plant-pathogen* models [54,55].

From the perspective of the pathogen, a single pathogenicity gene in *Zymoseptoria tritici*, G_07189 (an effector gene of avirulence), can elicit a spectrum of phenotypes in *T. aestivum*. Owing to the genetic diversity of the pathogen, the G_07189 gene can function as a classic avirulence gene as in the qualitative effect model [55], also known as the gene-by-gene model [56], or as a quantitative effect gene [55]. This behavior could hypothetically explain why individuals with transgressive resistance were observed for the Ang29 isolate but not for Cam1 or Que2 isolates. In this case, the virulence response of *C. kahawae* depends on its aggressiveness and/or geographic origin [34,35,36]. Therefore, the pathogenicity response of *C. kahawae*, such as resistance in *C. arabica*, is not a strictly quantitative or qualitative variable but is based on a continuum of diverse phenotypic responses, similar to that reported previously [55].

Similarly, for resistance exhibited by ET.56, the results are similar to that exhibited by Rume Sudan; that is, the resistance spans a continuum of phenotypic responses, and it can be concluded that the inheritance of resistance to *C. kahawae* derived from these two sources is due to the presence of genes with quantitative effects. Although there are differences between the populations derived from ET.56 and those derived from Rume Sudan, the dispersion of the curves of the cumulative percentage of hypocotyls with respect to susceptibility (Class 4) and the behavior of Ang29 and Que2 isolates by the donor sources of resistance exhibit very similar resistance percentages (Figure 2). They are probably due to similar genetic configurations; however, the presence of different resistance genes cannot be ruled out. If this were the case, the name *Ck-4* is proposed as a putative locus or part of a set of loci (QTLs) responsible for resistance to *C. kahawae* in the wild *C. arabica*, ET.56.

It is important to clarify that this name (*Ck-4*) is suggested and is based solely on the phenotypic evidence obtained, the segregation observed in the $F_{2}$ populations derived from ET.56 and the divergence of the cumulative percentage of susceptible hypocotyls (Class 4) to the Que2 isolate of *C. kahawae*. Therefore, for the assignment and formal confirmation of this locus or set of loci (*Ck-4*), as well as the confirmation of its differences from the *R* gene (*Ck-2* and *Ck-3*) in Rume Sudan [22], related genetic mapping and detailed molecular studies are needed.

### 4.3. Genetic Parameters Associated with Resistance to C. kahawae

A study [20] reported initial approximations of the type of inheritance of genes associated with resistance to *C. kahawae* in *C. arabica*, and another study [50] subsequently reported important observations in this context. Notably, in the first study, the interaction between genotypes and isolates was not considered, possibly for two reasons: (a) It was the first study of this type for the *C. arabica*/*C. kahawae* model; therefore, there was little information available for the *plant-pathogen* model, and (b) the research was directed toward other objectives. However, by not considering the G × I interaction in the analyses performed [20], the observed phenotypic response was attributed to genetic differences between the genotypes and uncontrolled variance. Therefore, the obtained heritability values of the resistance of *C. arabica* to *C. kahawae* (0.88–0.97) were possibly overestimated.

In this study, broad-sense heritability values of resistance to the *C. kahawae* isolate of Kenyan origin (Que2) were obtained. This parameter was derived from the analysis without considering the G × I interaction with values similar to those previously reported [20]. Despite the similarity of the reported heritability values (0.88–0.97) vs. the values observed (0.85–0.96) in this study, our quantitative analyses revealed that the G × I interaction is a factor that statistically contributes to the observed phenotypic resistance of *C. arabica* to *C. kahawae*. Therefore, the values that should be considered broad-sense heritability and that are most consistent with the phenotypic expression of resistance to *C. kahawae* observed are those derived from the genetic model that includes the G × I interaction (0.42–0.64). The heritability values of plants in terms of resistance to pathogens within this range have been documented (0.45 and 0.59) for the *T. aestivum*–*Z. tritici* model, for which the quantitative nature of plant resistance to the pathogen has been demonstrated [55].

However, the genetic interactions involved in the resistance of *C. arabica* to *C. kahawae* are complex, and it is possible that these interactions extend beyond those observed. The marked difference in the estimation of the heritability of resistance according to the crosses performed constitutes an important complementary finding. Notably, when the Rume Sudan variety was a female progenitor, the values of the genetic component and heritability ($H^{2}$) were higher (Table 7 and Table S14) than when it was a male parent. These differences hypothetically suggest the existence of maternal effects on the expression of resistance of *C. arabica* to *C. kahawae*.

Cytoplasmic effects on disease resistance have been demonstrated in *plant-pathogen* interaction models. For *T. aestivum*, maternal effects strongly contribute to resistance to *Z. tritici*, and in red clover, resistance to nodular bacteria is regulated by maternal inheritance [57,58] exclusively due to genes transmitted by the female progenitor. Additionally, it has been reported that resistance to *Mycosphaerella graminicola* in *T. aestivum* is conferred by cytoplasmic genes and that this resistance exhibits specificity (interaction); that is, it is effective only against certain isolates of *M. graminicola* [59].

The findings of these reports are consistent with the results obtained, specifically the highest heritability obtained when the Rume Sudan variety served as a female progenitor (0.642 vs. 0.423), suggesting that genetic factors of maternal origin are likely responsible for the expression of resistance to *C. kahawae*.

### 4.4. C. arabica/C. kahawae Interaction

Evidence of the existence of pathotypes of *C. kahawae* is scarce. The difficulty in their identification is due, among other factors, to the complexity of identifying *C. arabica* genotypes as differentials of possible virulent groups of *C. kahawae*. However, some efforts have already been made in this regard [6,42]. In the present investigation, the G × I interaction was significant (p<0.05). This finding confirms the presence of *C. arabica*/*C. kahawae* interactions and the existence of pathotypes in *C. kahawae*. However, the identification of strains of *C. kahawae* will be a challenge for genetic improvement programs aimed at resistance against CBD, as the results suggest that the genetic nature of resistance to the disease extends beyond the expression of genes with dominant effects.

### 4.5. Implications for the Breeding of C. arabica

Finally, this study provides new genetic evidence about a novel genetic source with high potential for *C. kahawae* resistance, a source that has thus far remained completely unexploited and unincorporated into the development of commercial cultivars of *C. arabica*. In light of these results, we strongly emphasize the strategic imperative of harnessing wild or underutilized genetic diversity in coffee germplasm banks.

These findings are of paramount importance for *C. arabica* breeding programs, providing the base foundation necessary to make highly informed, long-term decisions regarding the selection of parental sources of resistance. Furthermore, this result is crucial for designing appropriate breeding strategies aimed at developing *C. arabica* varieties with durable resistance against *C. kahawae*.

## 5. Conclusions

Traditionally, for imparting genetic resistance to CBD, the introgression of the Rume Sudan resistance gene *R*, assuming that it has a dominant effect, has been considered in genetic improvement programs of *C. arabica*. Contrary to its known resistance pattern, the present findings suggest that resistance to *C. kahawae* exhibited by Rume Sudan is polygenic and quantitative. The absence of repeatability in the expected theoretical segregation of each hybrid evaluated and the continuous phenotypic variation in the expression of resistance indicate that resistance to *C. kahawae* is due to genetic effects with different degrees of dominance. Therefore, resistance to *C. kahawae* cannot be explained only by a model of one gene (*R*) and its three genotypes (*RR*, *Rr*, and *rr*) for a segregating population. In contrast, it involves a more complex system of genes (QTLs) that act together.

Rume Sudan exhibited differences in heritability ($H^{2}$) based on whether it was used as a female ($H^{2}$ = 0.642) or a male ($H^{2}$ = 0.423) progenitor. This finding suggests the existence of cytoplasmic effects on the expression of resistance to *C. kahawae*, the confirmation of which requires complementary genetic studies. On the other hand, although the resistance of the $F_{2}$ populations derived from Rume Sudan and ET.56 is present within a continuum of phenotypic expressions, the differences in resistance to the isolate of Kenyan origin (Que2) indicate that the genetic configuration or the genetic mechanisms responsible for the resistance of Rume Sudan and ET.56 are likely different (*Ck-4*).

Aditionally, the significant values for the G × I interaction confirm the unequivocal existence of the *C. arabica*/*C. kahawae* interaction and demonstrate that resistance is not transversal but specific. This behavior is consistent with what is known about the aggressiveness of *C. kahawae*, which is dependent on its genetic diversity.

Finally, evidence is provided for a new source with potential resistance, a source thus far not incorporated in the development of commercial varieties of *C. arabica*; we emphasize the importance of using genetic diversity to address the outbreak of a potential disease such as CBD. These findings are important for breeding programs to make appropriate decisions about the sources of resistance and the strategy for the development of varieties of *C. arabica* with resistance to *C. kahawae*.

## Figures and Tables

**Table 1:** Populations evaluated and number of hypocotyls for each population.

CCC		Populations	NTH
Caturra	Commercial genotype	Susceptibility control to *C. kahawae*	724
SL.28	Commercial genotype	Susceptibility control to *C. kahawae*	353
Catuaí	Commercial genotype	Susceptibility progenitor to *C. kahawae*	403
Iapar-59	Commercial genotype	Susceptibility progenitor to *C. kahawae*	398
Rume Sudan	Wild genotype	Resistant progenitor to *C. kahawae*	174
ET.56	Wild genotype	Resistant progenitor to *C. kahawae*	179
2019_3 – 781 2019_3 – 782 2019_3 – 783 2019_3 – 784 2019_3 – 785 2019_3 – 786 2019_3 – 788 2019_3 – 791 2019_3 – 792 2019_3 – 793 2019_3 – 794 2019_3 – 795 2019_3 – 796	Iapar-59 × Rume Sudan	$F_{2}$ populations	4684
BGII – 667 BGII – 668 BGII – 669 BGII – 709 BGII – 724 BGII – 725 BGII – 867 BGII – 868 BGII – 875	Rume Sudan × Catuaí	$F_{2}$ populations	1623
2019_3 – 745 2019_3 – 746 2019_3 – 751 2019_3 – 752 2019_3 – 753 2019_3 – 754 2019_3 – 755 2019_3 – 758 2019_3 – 759	Iapar-59 × ET.56	$F_{2}$ populations	3873
NTH			12,411

CCC: Colombian Coffee Collection; BGII: Germplasm Bank Two; NTH: Total number of hypocotyls.

**Table 2:** Isolates of *C. kahawae* and their classification based on aggressiveness and geographic origin.

Origin	Isolates	Aggressiveness	References
Angola	Ang29	High	[35,36,37]
Cameroon	Cam1	High–Medium	[34,35,36,37]
Kenya	Que2	Medium–Low	[34,35,36,37]

**Table 3:** Scale for the classification of resistance of *C. arabica* to *C. kahawae* on the basis of the total percentage of hypocotyls exhibiting phenotypic resistance (Class 1 + Class 2 + Class 3).

Percentage of Resistant Hypocotyls	Resistance Class
>79%	High (HR)
>50% and ≤79%	Moderate Resistance (MR)
>20% and ≤50%	Low Resistance (LR)
>0% and ≤20%	Very Low Resistance (VLR)
=0%	Susceptible (S)

**Table 4:** Analysis of variance of the percentage of hypocotyls resistant to *C. kahawae* in segregating populations derived from Rume Sudan and ET.56.

Source	Factor	gl	SC	CM	F Value	Pr(>F)	Significance
	CCC (G)	29	4.740	0.163	2300	<0.001	***
	Isolate (I)	2	1.588	0.794	111.870	<0.001	***
Rume Sudan	G × I	34	0.880	0.026	3.680	<0.001	***
	Residuals	26	0.185	0.007			
	CCC (G)	12	2.341	0.195	24.799	<0.001	***
	Isolate (I)	2	0.674	0.337	42.818	<0.001	***
ET.56	G × I	23	0.507	0.022	2.802	0.013	*
	Residuals	19	0.149	0.008			

Significance level code α=0.05 (95% confidence): 0 ‘***’ 0.01 ‘*’.

**Table 5:** Mendelian segregation ratios (1:3, 3:1, 9:7, and 15:1) observed for the isolates Ang29, Cam1, and Que2 of *C. kahawae* across different populations.

			1:3		3:1		9:7		15:1	
Isolate	Population	n	✓	✗	✓	✗	✓	✗	✓	✗
	Iapar-59 × Rume Sudan	11	5	6	-	11	2	9	2	9
Ang29	Iapar-59 × ET.56	9	3	6	-	9	1	8	2	7
	Rume Sudan × Catuaí	3	1	2	-	3	2	1	-	3
	Iapar-59 × Rume Sudan	11	4	7	-	11	-	11	4	7
Cam1	Iapar-59 × ET.56	9	4	5	-	9	-	9	1	8
	Rume Sudan × Catuaí	1	-	1	-	1	-	1	-	1
	Iapar-59 × Rume Sudan	13	1	12	5	8	3	10	1	12
Que2	Iapar-59 × ET.56	9	-	9	-	9	5	4	-	9
	Rume Sudan × Catuaí	9	3	6	1	8	3	6	2	7

Segregation ratios calculated based on phenotypic responses. n: number of hybrids ($F_{1}$) evaluated and their respective segregating populations ($F_{2}$) per population; ✗: rejected (p<0.05); ✓: not significantly different from expectation (p≥0.05).

**Table 6:** Statistical values of the groups into which the isolates Ang29, Cam1, and Que2 of *C. kahawae* were divided according to aggressiveness and the genetic source, Rume Sudan or ET.56, from which they were derived.

Source	Isolate	% HR	*std*	*r*	*se*	LCL	UCL	Group
	Ang29	0.228	0.203	23	0.018	0.192	0.264	b
Rume Sudan	Cam1	0.172	0.164	19	0.019	0.132	0.211	b
	Que2	0.475	0.296	50	0.012	0.450	0.499	a
	Ang29	0.152	0.146	16	0.022	0.106	0.199	b
ET.56	Cam1	0.173	0.220	15	0.023	0.125	0.221	b
	Que2	0.412	0.268	26	0.017	0.375	0.448	a

%HR: Average percentage of resistant hypocotyls; std: standard deviation; *r*: number of repetitions; se: standard error; LCL: lower confidence limit; UCL: upper confidence limit. The same letters indicate that results do not differ statistically at a significance level of α=0.05 (95% confidence).

**Table 7:** Deduction of the parameters of the theoretical genetic model for the expression of resistance to *C. kahawae* in the segregating populations of *C. arabica* evaluated.

Population	$H^{2}$ (95% CI)	Genetic (%)	G × I (%)	Error (%)
Iapar-59 × Rume Sudan	0.423 (0.138–0.745)	42.3%	14.7%	7.4%
Rume Sudan × Catuaí	0.642 (0.194–0.867)	64.2%	25.1%	3.1%
Iapar-59 × ET.56	0.416 (0.119–0.730)	41.4%	11.9%	12.6%

$H^{2}$: broad-sense heritability; CI: confidence interval; Genetic (%): relative importance of the genetic component; G × I (%): relative importance of the genotype × isolate interaction component; Error (%): relative importance of the unexplained variance.

## Data Availability

Restrictions apply to the dataset. The data sets presented in this article are not easily available because they were obtained with resources from the National Federation of Coffee Growers of Colombia and are part of the development program for *C. arabica* varieties with genetic resistance to CBD. Requests to access the data sets should be directed to the National Coffee Research Center—Cenicafé.

## Supplementary Materials

The following supporting information can be downloaded from https://www.mdpi.com/article/10.3390/plants15132002/s1.
