# Supplementary material for: Genetic Control of Resistance to Colletotrichum kahawae in Coffee: Evidence of Polygenic Inheritance and Differential Host Genotype Responses to Pathogen Isolates
Source: Plants (Basel). 2026 Jun 28;15(13):2002. doi: 10.3390/plants15132002 (PMC13364353; doi:10.3390/plants15132002)
Supplement: Supplementary file 1 [file plants-15-02002-s001.zip › plants-4315752-supplementary.pdf]

Supplementary Materials

Supplementary Materials

Table S1. Chi-square goodness-of-fit test for the theoretical segregation of resistance (3:1) to the Ang29 isolate of *C. kahawae* based on the percentage of resistant hypocotyls of each F_2 population inoculated under controlled conditions.

CCC	Hybrids	NHRO	NHSO	NHRE	NHSE	NTH	*p* *value*	3:1
2019_3 – 781	Iapar-59 × Rume Sudan	9	93	76	26	102	< 0.050	✗
2019_3 – 782	Iapar-59 × Rume Sudan	27	72	74	25	99	< 0.050	✗
2019_3 – 783	Iapar-59 × Rume Sudan	35	67	76	26	102	< 0.050	✗
2019_3 – 784	Iapar-59 × Rume Sudan	12	101	84	29	113	< 0.050	✗
2019_3 – 785	Iapar-59 × Rume Sudan	26	66	69	23	92	< 0.050	✗
2019_3 – 786	Iapar-59 × Rume Sudan	34	72	79	27	106	< 0.050	✗
2019_3 – 788	Iapar-59 × Rume Sudan	43	59	76	26	102	< 0.050	✗
2019_3 – 791	Iapar-59 × Rume Sudan	19	83	76	26	102	< 0.050	✗
2019_3 – 792	Iapar-59 × Rume Sudan	18	83	75	26	101	< 0.050	✗
2019_3 – 795	Iapar-59 × Rume Sudan	47	47	70	24	94	< 0.050	✗
2019_3 – 796	Iapar-59 × Rume Sudan	54	47	75	26	101	< 0.050	✗
2019_3 – 745	Iapar-59 × ET.56	25	180	153	52	205	< 0.050	✗
2019_3 – 746	Iapar-59 × ET.56	10	110	90	30	120	< 0.050	✗
2019_3 – 751	Iapar-59 × ET.56	30	77	80	27	107	< 0.050	✗
2019_3 – 752	Iapar-59 × ET.56	8	97	78	27	105	< 0.050	✗
2019_3 – 753	Iapar-59 × ET.56	23	37	45	15	60	< 0.050	✗
2019_3 – 754	Iapar-59 × ET.56	22	81	77	26	103	< 0.050	✗
2019_3 – 755	Iapar-59 × ET.56	11	89	75	25	100	< 0.050	✗
2019_3 – 758	Iapar-59 × ET.56	28	60	66	22	88	< 0.050	✗
2019_3 – 759	Iapar-59 × ET.56	41	54	71	24	95	< 0.050	✗
BGII – 667	Rume Sudan × Catuaí	58	45	77	26	103	< 0.050	✗
BGII – 868	Rume Sudan × Catuaí	45	26	53	18	71	< 0.050	✗
BGII – 875	Rume Sudan × Catuaí	16	33	36	13	49	< 0.050	✗

For $\alpha = 0.05$ (95% confidence); $gl = 1$, and the critical value is 3.841. *CCC*: Colombian Coffee Collection; *BGII*: Germplasm Bank Two; *NHRO*; Observed resistant hypocotyls; *NHSO*: Observed Susceptible Hypocotyls; *NHRE*; Expected Resistant Hypocotyls; *NHSE*: Expected Susceptible Hypocotyls; *NTH*: Total Number of Hypocotyls; ✗: rejected ($p < 0.05$); ✓: not significantly different from expectation ($p \geq 0.05$).

Table S2. Chi-square goodness-of-fit test for the theoretical segregation of resistance (1:3) to the Ang29 isolate of *C. kahawae* based on the percentage of resistant hypocotyls of each F_2 population inoculated under controlled conditions.

CCC	Hybrids	NHRO	NHSO	NHRE	NHSE	NTH	*p value*	1:3
2019_3 – 781	Iapar-59 × Rume Sudan	9	93	26	76	102	< 0.050	✗
2019_3 – 782	Iapar-59 × Rume Sudan	27	72	25	74	99	0.601	✓
2019_3 – 783	Iapar-59 × Rume Sudan	35	67	26	76	102	< 0.050	✗
2019_3 – 784	Iapar-59 × Rume Sudan	12	101	29	84	113	< 0.050	✗
2019_3 – 785	Iapar-59 × Rume Sudan	26	66	23	69	92	0.470	✓
2019_3 – 786	Iapar-59 × Rume Sudan	34	72	27	79	106	0.092	✓
2019_3 – 788	Iapar-59 × Rume Sudan	43	59	26	76	102	< 0.050	✗
2019_3 – 791	Iapar-59 × Rume Sudan	19	83	26	76	102	0.137	✓
2019_3 – 792	Iapar-59 × Rume Sudan	18	83	26	75	101	0.095	✓
2019_3 – 795	Iapar-59 × Rume Sudan	47	47	24	70	94	< 0.050	✗
2019_3 – 796	Iapar-59 × Rume Sudan	54	47	26	75	101	< 0.050	✗
2019_3 – 745	Iapar-59 × ET.56	25	180	52	153	205	< 0.050	✗
2019_3 – 746	Iapar-59 × ET.56	10	110	30	90	120	< 0.050	✗
2019_3 – 751	Iapar-59 × ET.56	30	77	27	80	107	0.468	✓
2019_3 – 752	Iapar-59 × ET.56	8	97	27	78	105	< 0.050	✗
2019_3 – 753	Iapar-59 × ET.56	23	37	15	45	60	< 0.050	✗
2019_3 – 754	Iapar-59 × ET.56	22	81	26	77	103	0.393	✓
2019_3 – 755	Iapar-59 × ET.56	11	89	25	75	100	< 0.050	✗
2019_3 – 758	Iapar-59 × ET.57	28	60	22	66	88	0.139	✓
2019_3 – 759	Iapar-59 × ET.56	41	54	24	71	95	< 0.050	✗
BGII – 667	Rume Sudan × Catuaí	58	45	26	77	103	< 0.050	✗
BGII – 868	Rume Sudan × Catuaí	45	26	18	53	71	< 0.050	✗
BGII – 875	Rume Sudan × Catuaí	16	33	13	36	49	0.216	✓

For $\alpha = 0.05$ (95% confidence); *gl* = 1, and the critical value is 3.841. *CCC*: Colombian Coffee Collection; *BGII*: Germplasm Bank Two; *NHRO*; Observed resistant hypocotyls; *NHSO*: Observed Susceptible Hypocotyls; *NHRE*; Expected Resistant Hypocotyls; *NHSE*: Expected Susceptible Hypocotyls; *NTH*: Total Number of Hypocotyls; ✗: rejected ($p < 0.05$); ✓: not significantly different from expectation ($p \geq 0.05$).

Table S3. Chi-square goodness-of-fit test for the theoretical segregation of resistance (9:7) of the Ang29 isolate to *C. kahawae* based on the percentage of resistant hypocotyls of each F_2 population inoculated under controlled conditions.

CCC	Hybrids	NHRO	NHSO	NHRE	NHSE	NTH	*p value*	9:7
2019_3 – 781	Iapar-59 × Rume Sudan	9	93	58	44	102	< 0.050	✗
2019_3 – 782	Iapar-59 × Rume Sudan	27	72	56	43	99	< 0.050	✗
2019_3 – 783	Iapar-59 × Rume Sudan	35	67	58	44	102	< 0.050	✗
2019_3 – 784	Iapar-59 × Rume Sudan	12	101	64	49	113	< 0.050	✗
2019_3 – 785	Iapar-59 × Rume Sudan	26	66	52	40	92	< 0.050	✗
2019_3 – 786	Iapar-59 × Rume Sudan	34	72	60	46	106	< 0.050	✗
2019_3 – 788	Iapar-59 × Rume Sudan	43	59	58	44	102	0.041	✗
2019_3 – 791	Iapar-59 × Rume Sudan	19	83	58	44	102	< 0.050	✗
2019_3 – 792	Iapar-59 × Rume Sudan	18	83	57	44	101	< 0.050	✗
2019_3 – 795	Iapar-59 × Rume Sudan	47	47	53	41	94	0.684	✓
2019_3 – 796	Iapar-59 × Rume Sudan	54	47	57	44	101	0.957	✓
2019_3 – 745	Iapar-59 × ET.56	25	180	116	89	205	< 0.050	✗
2019_3 – 746	Iapar-59 × ET.56	10	110	68	52	120	< 0.050	✗
2019_3 – 751	Iapar-59 × ET.56	30	77	61	46	107	< 0.050	✗
2019_3 – 752	Iapar-59 × ET.56	8	97	60	45	105	< 0.050	✗
2019_3 – 753	Iapar-59 × ET.56	23	37	34	26	60	0.049	✗
2019_3 – 754	Iapar-59 × ET.56	22	81	58	45	103	< 0.050	✗
2019_3 – 755	Iapar-59 × ET.56	11	89	57	43	100	< 0.050	✗
2019_3 – 758	Iapar-59 × ET.56	28	60	50	38	88	< 0.050	✗
2019_3 – 759	Iapar-59 × ET.56	41	54	54	41	95	0.085	✓
BGII – 667	Rume Sudan × Catuaí	58	45	58	45	103	1.000	✓
BGII – 868	Rume Sudan × Catuaí	45	26	40	31	71	0.690	✓
BGII – 875	Rume Sudan × Catuaí	16	33	28	21	49	0.011	✗

For $\alpha = 0.05$ (95% confidence); *gl* = 1, and the critical value is 3.841. *CCC*: Colombian Coffee Collection; *BGII*: Germplasm Bank Two; *NHRO*; Observed resistant hypocotyls; *NHSO*: Observed Susceptible Hypocotyls; *NHRE*; Expected Resistant Hypocotyls; *NHSE*: Expected Susceptible Hypocotyls; *NTH*: Total Number of Hypocotyls; ✗: rejected ($p < 0.05$); ✓: not significantly different from expectation ($p \geq 0.05$).

Table S4. Chi-square goodness-of-fit test for the theoretical segregation of resistance (15:1) to the Ang29 isolate of *C. kahawae* based on the percentage of resistant hypocotyls of each F_2 population inoculated under controlled conditions.

CCC	Hybrids	NHRO	NHSO	NHRE	NHSE	NTH	*p value*	15:1
2019_3 – 781	Iapar-59 × Rume Sudan	9	93	96	6	102	0.282	✓
2019_3 – 782	Iapar-59 × Rume Sudan	27	72	93	6	99	< 0.050	✗
2019_3 – 783	Iapar-59 × Rume Sudan	35	67	96	6	102	< 0.050	✗
2019_3 – 784	Iapar-59 × Rume Sudan	12	101	106	7	113	0.055	✓
2019_3 – 785	Iapar-59 × Rume Sudan	26	66	87	5	92	< 0.050	✗
2019_3 – 786	Iapar-59 × Rume Sudan	34	72	100	6	106	< 0.050	✗
2019_3 – 788	Iapar-59 × Rume Sudan	43	59	96	6	102	< 0.050	✗
2019_3 – 791	Iapar-59 × Rume Sudan	19	83	96	6	102	< 0.050	✗
2019_3 – 792	Iapar-59 × Rume Sudan	18	83	95	6	101	< 0.050	✗
2019_3 – 795	Iapar-59 × Rume Sudan	47	47	89	5	94	< 0.050	✗
2019_3 – 796	Iapar-59 × Rume Sudan	54	47	95	6	101	< 0.050	✗
2019_3 – 745	Iapar-59 × ET.56	25	180	193	12	205	< 0.050	✗
2019_3 – 746	Iapar-59 × ET.56	10	110	113	7	120	0.345	✓
2019_3 – 751	Iapar-59 × ET.56	30	77	101	6	107	< 0.050	✗
2019_3 – 752	Iapar-59 × ET.56	8	97	99	6	105	0.562	✓
2019_3 – 753	Iapar-59 × ET.56	23	37	57	3	60	< 0.050	✗
2019_3 – 754	Iapar-59 × ET.56	22	81	97	6	103	< 0.050	✗
2019_3 – 755	Iapar-59 × ET.56	11	89	94	6	100	< 0.050	✗
2019_3 – 758	Iapar-59 × ET.56	28	60	83	5	88	< 0.050	✗
2019_3 – 759	Iapar-59 × ET.56	41	54	90	5	95	< 0.050	✗
BGII – 667	Rume Sudan × Catuaí	58	45	97	6	103	< 0.050	✗
BGII – 868	Rume Sudan × Catuaí	45	26	67	4	71	< 0.050	✗
BGII – 875	Rume Sudan × Catuaí	16	33	46	3	49	< 0.050	✗

For $\alpha = 0.05$ (95% confidence); *gl* = 1, and the critical value is 3.841. *CCC*: Colombian Coffee Collection; *BGII*: Germplasm Bank Two; *NHRO*; Observed resistant hypocotyls; *NHSO*: Observed Susceptible Hypocotyls; *NHRE*; Expected Resistant Hypocotyls; *NHSE*: Expected Susceptible Hypocotyls; *NTH*: Total Number of Hypocotyls; ✗: rejected ($p < 0.05$); ✓: not significantly different from expectation ($p \geq 0.05$).

Table S5. Chi-square goodness-of-fit test for the theoretical segregation of resistance (3:1) to the Cam1 isolate of *C. kahawae* based on the percentage of resistant hypocotyls of each F_2 population inoculated under controlled conditions.

CCC	Hybrids	NHRO	NHSO	NHRE	NHSE	NTH	*p value*	3:1
2019_3 – 781	Iapar-59 × Rume Sudan	10	96	79	27	106	< 0.050	✗
2019_3 – 782	Iapar-59 × Rume Sudan	41	70	83	28	111	< 0.050	✗
2019_3 – 783	Iapar-59 × Rume Sudan	24	70	70	24	94	< 0.050	✗
2019_3 – 784	Iapar-59 × Rume Sudan	7	96	77	26	103	< 0.050	✗
2019_3 – 785	Iapar-59 × Rume Sudan	10	83	69	24	93	< 0.050	✗
2019_3 – 786	Iapar-59 × Rume Sudan	23	42	48	17	65	< 0.050	✗
2019_3 – 788	Iapar-59 × Rume Sudan	12	77	66	23	89	< 0.050	✗
2019_3 – 791	Iapar-59 × Rume Sudan	5	52	42	15	57	< 0.050	✗
2019_3 – 792	Iapar-59 × Rume Sudan	1	103	78	26	104	< 0.050	✗
2019_3 – 795	Iapar-59 × Rume Sudan	34	73	80	27	107	< 0.050	✗
2019_3 – 796	Iapar-59 × Rume Sudan	27	55	61	21	82	< 0.050	✗
2019_3 – 745	Iapar-59 × ET.56	26	146	129	43	172	< 0.050	✗
2019_3 – 746	Iapar-59 × ET.56	12	99	83	28	111	< 0.050	✗
2019_3 – 751	Iapar-59 × ET.56	21	68	66	23	89	< 0.050	✗
2019_3 – 752	Iapar-59 × ET.56	32	79	83	28	111	< 0.050	✗
2019_3 – 753	Iapar-59 × ET.56	25	78	77	26	103	< 0.050	✗
2019_3 – 754	Iapar-59 × ET.56	6	86	69	23	92	< 0.050	✗
2019_3 – 755	Iapar-59 × ET.56	15	90	78	27	105	< 0.050	✗
2019_3 – 758	Iapar-59 × ET.56	14	89	77	26	103	< 0.050	✗
2019_3 – 759	Iapar-59 × ET.56	26	84	82	28	110	< 0.050	✗
BGII – 667	Rume Sudan × Catuaí	11	68	59	20	79	< 0.050	✗

For $\alpha = 0.05$ (95% confidence); *gl* = 1, and the critical value is 3.841. *CCC*: Colombian Coffee Collection; *BGII*: Germplasm Bank Two; *NHRO*; Observed resistant hypocotyls; *NHSO*: Observed Susceptible Hypocotyls; *NHRE*; Expected Resistant Hypocotyls; *NHSE*: Expected Susceptible Hypocotyls; *NTH*: Total Number of Hypocotyls; ✗: rejected ($p < 0.05$); ✓: not significantly different from expectation ($p \geq 0.05$).

Table S6. Chi-square goodness-of-fit test for the theoretical segregation of resistance (1:3) to the Cam1 isolate of *C. kahawae* based on the percentage of resistant hypocotyls of each F_2 population inoculated under controlled conditions.

CCC	Hybrids	NHRO	NHSO	NHRE	NHSE	NTH	*p value*	1:3
2019_3 – 781	Iapar-59 × Rume Sudan	10	96	27	79	106	< 0.050	✗
2019_3 – 782	Iapar-59 × Rume Sudan	41	70	28	83	111	< 0.050	✗
2019_3 – 783	Iapar-59 × Rume Sudan	24	70	24	70	94	0.905	✓
2019_3 – 784	Iapar-59 × Rume Sudan	7	96	26	77	103	< 0.050	✗
2019_3 – 785	Iapar-59 × Rume Sudan	10	83	24	69	93	< 0.050	✗
2019_3 – 786	Iapar-59 × Rume Sudan	23	42	17	48	65	0.053	✓
2019_3 – 788	Iapar-59 × Rume Sudan	12	77	23	66	89	< 0.050	✗
2019_3 – 791	Iapar-59 × Rume Sudan	5	52	15	42	57	< 0.050	✗
2019_3 – 792	Iapar-59 × Rume Sudan	1	103	26	78	104	< 0.050	✗
2019_3 – 795	Iapar-59 × Rume Sudan	34	73	27	80	107	0.105	✓
2019_3 – 796	Iapar-59 × Rume Sudan	27	55	21	61	82	0.097	✓
2019_3 – 745	Iapar-59 × ET.56	26	146	43	129	172	< 0.050	✗
2019_3 – 746	Iapar-59 × ET.56	12	99	28	83	111	< 0.050	✗
2019_3 – 751	Iapar-59 × ET.56	21	68	23	66	89	0.759	✓
2019_3 – 752	Iapar-59 × ET.56	32	79	28	83	111	0.351	✓
2019_3 – 753	Iapar-59 × ET.56	25	78	26	77	103	0.864	✓
2019_3 – 754	Iapar-59 × ET.56	6	86	23	69	92	< 0.050	✗
2019_3 – 755	Iapar-59 × ET.56	15	90	27	78	105	< 0.050	✗
2019_3 – 758	Iapar-59 × ET.56	14	89	26	77	103	< 0.050	✗
2019_3 – 759	Iapar-59 × ET.56	26	84	28	82	110	0.741	✓
BGII – 667	Rume Sudan × Catuaí	11	68	20	59	79	< 0.050	✗

For $\alpha = 0.05$ (95% confidence); *gl* = 1, and the critical value is 3.841. *CCC*: Colombian Coffee Collection; *BGII*: Germplasm Bank Two; *NHRO*; Observed resistant hypocotyls; *NHSO*: Observed Susceptible Hypocotyls; *NHRE*; Expected Resistant Hypocotyls; *NHSE*: Expected Susceptible Hypocotyls; *NTH*: Total Number of Hypocotyls; ✗: rejected ($p < 0.05$); ✓: not significantly different from expectation ($p \geq 0.05$).

Table S7. Chi-square goodness-of-fit test for the theoretical segregation of resistance (9:7) to the Cam1 isolate of *C. kahawae* based on the percentage of resistant hypocotyls of each F_2 population inoculated under controlled conditions.

CCC	Hybrids	NHRO	NHSO	NHRE	NHSE	NTH	*p value*	9:7
2019_3 – 781	Iapar-59 × Rume Sudan	10	96	60	46	106	< 0.050	✗
2019_3 – 782	Iapar-59 × Rume Sudan	41	70	63	48	111	< 0.050	✗
2019_3 – 783	Iapar-59 × Rume Sudan	24	70	53	41	94	< 0.050	✗
2019_3 – 784	Iapar-59 × Rume Sudan	7	96	58	45	103	< 0.050	✗
2019_3 – 785	Iapar-59 × Rume Sudan	10	83	53	40	93	< 0.050	✗
2019_3 – 786	Iapar-59 × Rume Sudan	23	42	37	28	65	< 0.050	✗
2019_3 – 788	Iapar-59 × Rume Sudan	12	77	51	38	89	< 0.050	✗
2019_3 – 791	Iapar-59 × Rume Sudan	5	52	33	24	57	< 0.050	✗
2019_3 – 792	Iapar-59 × Rume Sudan	1	103	59	45	104	< 0.050	✗
2019_3 – 795	Iapar-59 × Rume Sudan	34	73	61	46	107	< 0.050	✗
2019_3 – 796	Iapar-59 × Rume Sudan	27	55	47	35	82	< 0.050	✗
2019_3 – 745	Iapar-59 × ET.56	26	146	97	75	172	< 0.050	✗
2019_3 – 746	Iapar-59 × ET.56	12	99	63	48	111	< 0.050	✗
2019_3 – 751	Iapar-59 × ET.56	21	68	51	38	89	< 0.050	✗
2019_3 – 752	Iapar-59 × ET.56	32	79	63	48	111	< 0.050	✗
2019_3 – 753	Iapar-59 × ET.56	25	78	58	45	103	< 0.050	✗
2019_3 – 754	Iapar-59 × ET.56	6	86	52	40	92	< 0.050	✗
2019_3 – 755	Iapar-59 × ET.56	15	90	60	45	105	< 0.050	✗
2019_3 – 758	Iapar-59 × ET.56	14	89	58	45	103	< 0.050	✗
2019_3 – 759	Iapar-59 × ET.56	26	84	62	48	110	< 0.050	✗
BGII – 667	Rume Sudan × Catuaí	11	68	45	34	79	< 0.050	✗

For $\alpha = 0.05$ (95% confidence); *gl* = 1, and the critical value is 3.841. *CCC*: Colombian Coffee Collection; *BGII*: Germplasm Bank Two; *NHRO*; Observed resistant hypocotyls; *NHSO*: Observed Susceptible Hypocotyls; *NHRE*; Expected Resistant Hypocotyls; *NHSE*: Expected Susceptible Hypocotyls; *NTH*: Total Number of Hypocotyls; ✗: rejected ($p < 0.05$); ✓: not significantly different from expectation ($p \geq 0.05$).

Table S8. Chi-square goodness-of-fit test for the theoretical segregation of resistance (15:1) to the Cam1 isolate of *C. kahawae* based on the percentage of resistant hypocotyls of each F_2 population inoculated under controlled conditions.

CCC	Hybrids	NHRO	NHSO	NHRE	NHSE	NTH	*p value*	15:1
2019_3 – 781	Iapar-59 × Rume Sudan	10	96	100	6	106	0.175	✓
2019_3 – 782	Iapar-59 × Rume Sudan	41	70	105	6	111	< 0.050	✗
2019_3 – 783	Iapar-59 × Rume Sudan	24	70	89	5	94	< 0.050	✗
2019_3 – 784	Iapar-59 × Rume Sudan	7	96	97	6	103	0.818	✓
2019_3 – 785	Iapar-59 × Rume Sudan	10	83	88	5	93	0.072	✓
2019_3 – 786	Iapar-59 × Rume Sudan	23	42	61	4	65	< 0.050	✗
2019_3 – 788	Iapar-59 × Rume Sudan	12	77	84	5	89	< 0.050	✗
2019_3 – 791	Iapar-59 × Rume Sudan	5	52	54	3	57	0.431	✓
2019_3 – 792	Iapar-59 × Rume Sudan	1	103	98	6	104	< 0.050	✗
2019_3 – 795	Iapar-59 × Rume Sudan	34	73	101	6	107	< 0.050	✗
2019_3 – 796	Iapar-59 × Rume Sudan	27	55	77	5	82	< 0.050	✗
2019_3 – 745	Iapar-59 × ET.56	26	146	162	10	172	< 0.050	✗
2019_3 – 746	Iapar-59 × ET.56	12	99	105	6	111	< 0.050	✗
2019_3 – 751	Iapar-59 × ET.56	21	68	84	5	89	< 0.050	✗
2019_3 – 752	Iapar-59 × ET.56	32	79	105	6	111	< 0.050	✗
2019_3 – 753	Iapar-59 × ET.56	25	78	97	6	103	< 0.050	✗
2019_3 – 754	Iapar-59 × ET.56	6	86	87	5	92	0.914	✓
2019_3 – 755	Iapar-59 × ET.56	15	90	99	6	105	< 0.050	✗
2019_3 – 758	Iapar-59 × ET.56	14	89	97	6	103	< 0.050	✗
2019_3 – 759	Iapar-59 × ET.56	26	84	104	6	110	< 0.050	✗
BGII – 667	Rume Sudan × Catuaí	11	68	75	4	79	< 0.050	✗

For $\alpha = 0.05$ (95% confidence); $gl = 1$, and the critical value is 3.841. *CCC*: Colombian Coffee Collection; *BGII*: Germplasm Bank Two; *NHRO*; Observed resistant hypocotyls; *NHSO*: Observed Susceptible Hypocotyls; *NHRE*; Expected Resistant Hypocotyls; *NHSE*: Expected Susceptible Hypocotyls; *NTH*: Total Number of Hypocotyls; ✗: rejected ($p < 0.05$); ✓: not significantly different from expectation ($p \geq 0.05$).

Table S9. Chi-square goodness-of-fit test for the theoretical segregation of resistance (3:1) to the Que2 isolate of *C. kahawae* based on the percentage of resistant hypocotyls of each F_2 population inoculated under controlled conditions.

CCC	Hybrids	NHRO	NHSO	NHRE	NHSE	NTH	*p value*	3:1
2019_3 – 781	Iapar-59 × Rume Sudan	179	223	301	101	402	< 0.050	✗
2019_3 – 782	Iapar-59 × Rume Sudan	143	51	145	49	194	0.678	✓
2019_3 – 783	Iapar-59 × Rume Sudan	107	22	96	33	129	< 0.050	✗
2019_3 – 784	Iapar-59 × Rume Sudan	119	86	153	52	205	< 0.050	✗
2019_3 – 785	Iapar-59 × Rume Sudan	114	29	107	36	143	0.192	✓
2019_3 – 786	Iapar-59 × Rume Sudan	153	56	156	53	209	0.549	✓
2019_3 – 788	Iapar-59 × Rume Sudan	140	62	151	51	202	0.061	✓
2019_3 – 791	Iapar-59 × Rume Sudan	87	130	162	55	217	< 0.050	✗
2019_3 – 792	Iapar-59 × Rume Sudan	103	100	152	51	203	< 0.050	✗
2019_3 – 795	Iapar-59 × Rume Sudan	111	85	147	49	196	< 0.050	✗
2019_3 – 796	Iapar-59 × Rume Sudan	151	52	152	51	203	0.839	✓
2019_3 – 793	Iapar-59 × Rume Sudan	19	82	75	26	101	< 0.050	✗
2019_3 – 794	Iapar-59 × Rume Sudan	12	143	116	39	155	< 0.050	✗
2019_3 – 745	Iapar-59 × ET.56	145	207	264	88	352	< 0.050	✗
2019_3 – 746	Iapar-59 × ET.56	91	132	167	56	223	< 0.050	✗
2019_3 – 751	Iapar-59 × ET.56	115	95	157	53	210	< 0.050	✗
2019_3 – 752	Iapar-59 × ET.56	74	113	140	47	187	< 0.050	✗
2019_3 – 753	Iapar-59 × ET.56	53	34	65	22	87	< 0.050	✗
2019_3 – 754	Iapar-59 × ET.56	105	123	171	57	228	< 0.050	✗
2019_3 – 755	Iapar-59 × ET.56	101	99	150	50	200	< 0.050	✗
2019_3 – 758	Iapar-59 × ET.56	121	96	162	55	217	< 0.050	✗
2019_3 – 759	Iapar-59 × ET.56	120	70	142	48	190	< 0.050	✗
BGII – 667	Rume Sudan × Catuaí	203	157	270	90	360	< 0.050	✗
BGII – 867	Rume Sudan × Catuaí	42	24	49	17	66	< 0.050	✗
BGII – 868	Rume Sudan × Catuaí	132	57	141	48	189	0.101	✓
BGII – 875	Rume Sudan × Catuaí	71	44	86	29	115	< 0.050	✗
BGII – 724	Rume Sudan × Catuaí	30	106	102	34	136	< 0.050	✗
BGII – 709	Rume Sudan × Catuaí	28	82	82	28	110	< 0.050	✗
BGII – 668	Rume Sudan × Catuaí	9	101	82	28	110	< 0.050	✗
BGII – 725	Rume Sudan × Catuaí	25	75	75	25	100	< 0.050	✗
BGII – 669	Rume Sudan × Catuaí	4	101	78	27	105	< 0.050	✗

For $\alpha = 0.05$ (95% confidence); gl = 1, and the critical value is 3.841. CCC: Colombian Coffee Collection; $BGII$: Germplasm Bank Two; $NHRO$; Observed resistant hypocotyls; $NHSO$: Observed Susceptible Hypocotyls; $NHRE$; Expected Resistant Hypocotyls; $NHSE$: Expected Susceptible Hypocotyls; NTH: Total Number of Hypocotyls; ✗: rejected ($p < 0.05$); ✓: not significantly different from expectation ($p \geq 0.05$).

Table S10. Chi-square goodness-of-fit test for the theoretical segregation of resistance (1:3) to the Que2 isolate of *C. kahawae* based on the percentage of resistant hypocotyls of each F_2 population inoculated under controlled conditions.

CCC	Hybrids	NHRO	NHSO	NHRE	NHSE	NTH	*p* *value*	1:3
2019_3 – 781	Iapar-59 × Rume Sudan	179	223	101	301	402	< 0.050	✗
2019_3 – 782	Iapar-59 × Rume Sudan	143	51	49	145	194	< 0.050	✗
2019_3 – 783	Iapar-59 × Rume Sudan	107	22	33	96	129	< 0.050	✗
2019_3 – 784	Iapar-59 × Rume Sudan	119	86	52	153	205	< 0.050	✗
2019_3 – 785	Iapar-59 × Rume Sudan	114	29	36	107	143	< 0.050	✗
2019_3 – 786	Iapar-59 × Rume Sudan	153	56	53	156	209	< 0.050	✗
2019_3 – 788	Iapar-59 × Rume Sudan	140	62	51	151	202	< 0.050	✗
2019_3 – 791	Iapar-59 × Rume Sudan	87	130	55	162	217	< 0.050	✗
2019_3 – 792	Iapar-59 × Rume Sudan	103	100	51	152	203	< 0.050	✗
2019_3 – 795	Iapar-59 × Rume Sudan	111	85	49	147	196	< 0.050	✗
2019_3 – 796	Iapar-59 × Rume Sudan	151	52	51	152	203	< 0.050	✗
2019_3 – 793	Iapar-59 × Rume Sudan	19	82	26	75	101	0.150	✓
2019_3 – 794	Iapar-59 × Rume Sudan	12	143	39	116	155	< 0.050	✗
2019_3 – 745	Iapar-59 × ET.56	145	207	88	264	352	< 0.050	✗
2019_3 – 746	Iapar-59 × ET.56	91	132	56	167	223	< 0.050	✗
2019_3 – 751	Iapar-59 × ET.56	115	95	53	157	210	< 0.050	✗
2019_3 – 752	Iapar-59 × ET.56	74	113	47	140	187	< 0.050	✗
2019_3 – 753	Iapar-59 × ET.56	53	34	22	65	87	< 0.050	✗
2019_3 – 754	Iapar-59 × ET.56	105	123	57	171	228	< 0.050	✗
2019_3 – 755	Iapar-59 × ET.56	101	99	50	150	200	< 0.050	✗
2019_3 – 758	Iapar-59 × ET.56	121	96	55	162	217	< 0.050	✗
2019_3 – 759	Iapar-59 × ET.56	120	70	48	142	190	< 0.050	✗
BGII – 667	Rume Sudan × Catuaí	203	157	90	270	360	< 0.050	✗
BGII – 867	Rume Sudan × Catuaí	42	24	17	49	66	< 0.050	✗
BGII – 868	Rume Sudan × Catuaí	132	57	48	141	189	< 0.050	✗
BGII – 875	Rume Sudan × Catuaí	71	44	29	86	115	< 0.050	✗
BGII – 724	Rume Sudan × Catuaí	30	106	34	102	136	0.428	✓
BGII – 709	Rume Sudan × Catuaí	28	82	28	82	110	0.912	✓
BGII – 668	Rume Sudan × Catuaí	9	101	28	82	110	< 0.050	✗
BGII – 725	Rume Sudan × Catuaí	25	75	25	75	100	1.000	✓
BGII – 669	Rume Sudan × Catuaí	4	101	27	78	105	< 0.050	✗

For $\alpha = 0.05$ (95% confidence); gl = 1, and the critical value is 3.841. CCC: Colombian Coffee Collection; $BGII$: Germplasm Bank Two; $NHRO$; Observed resistant hypocotyls; $NHSO$: Observed Susceptible Hypocotyls; $NHRE$; Expected Resistant Hypocotyls; $NHSE$: Expected Susceptible Hypocotyls; NTH: Total Number of Hypocotyls; ✗: rejected ($p < 0.05$); ✓: not significantly different from expectation ($p \geq 0.05$).

Table S11. Chi-square goodness-of-fit test for the theoretical segregation of resistance (9:7) to the Que2 isolate of *C. kahawae* based on the percentage of resistant hypocotyls of each F_2 population inoculated under controlled conditions.

CCC	Hybrids	NHRO	NHSO	NHRE	NHSE	NTH	*p value*	9:7
2019_3 – 781	Iapar-59 × Rume Sudan	179	223	227	175	402	< 0.050	✗
2019_3 – 782	Iapar-59 × Rume Sudan	143	51	110	84	194	< 0.050	✗
2019_3 – 783	Iapar-59 × Rume Sudan	107	22	73	56	129	< 0.050	✗
2019_3 – 784	Iapar-59 × Rume Sudan	119	86	193	12	205	0.966	✓
2019_3 – 785	Iapar-59 × Rume Sudan	114	29	135	8	143	< 0.050	✗
2019_3 – 786	Iapar-59 × Rume Sudan	153	56	196	13	209	< 0.050	✗
2019_3 – 788	Iapar-59 × Rume Sudan	140	62	190	12	202	< 0.050	✗
2019_3 – 791	Iapar-59 × Rume Sudan	87	130	204	13	217	< 0.050	✗
2019_3 – 792	Iapar-59 × Rume Sudan	103	100	191	12	203	0.474	✓
2019_3 – 795	Iapar-59 × Rume Sudan	111	85	184	12	196	0.999	✓
2019_3 – 796	Iapar-59 × Rume Sudan	151	52	191	12	203	< 0.050	✗
2019_3 – 793	Iapar-59 × Rume Sudan	19	82	95	6	101	< 0.050	✗
2019_3 – 794	Iapar-59 × Rume Sudan	12	143	146	9	155	< 0.050	✗
2019_3 – 745	Iapar-59 × ET.56	145	207	330	22	352	< 0.050	✗
2019_3 – 746	Iapar-59 × ET.56	91	132	210	13	223	< 0.050	✗
2019_3 – 751	Iapar-59 × ET.56	115	95	197	13	210	0.979	✓
2019_3 – 752	Iapar-59 × ET.56	74	113	176	11	187	< 0.050	✗
2019_3 – 753	Iapar-59 × ET.56	53	34	82	5	87	0.856	✓
2019_3 – 754	Iapar-59 × ET.56	105	123	214	14	228	< 0.050	✗
2019_3 – 755	Iapar-59 × ET.56	101	99	188	12	200	0.442	✓
2019_3 – 758	Iapar-59 × ET.56	121	96	204	13	217	0.999	✓
2019_3 – 759	Iapar-59 × ET.56	120	70	179	11	190	0.297	✓
BGII – 667	Rume Sudan × Catuaí	203	157	338	22	360	0.999	✓
BGII – 867	Rume Sudan × Catuaí	42	24	62	4	66	0.690	✓
BGII – 868	Rume Sudan × Catuaí	132	57	178	11	189	< 0.050	✗
BGII – 875	Rume Sudan × Catuaí	71	44	108	7	115	0.703	✓
BGII – 724	Rume Sudan × Catuaí	30	106	128	8	136	< 0.050	✗
BGII – 709	Rume Sudan × Catuaí	28	82	104	6	110	< 0.050	✗
BGII – 668	Rume Sudan × Catuaí	9	101	104	6	110	< 0.050	✗
BGII – 725	Rume Sudan × Catuaí	25	75	94	6	100	< 0.050	✗
BGII – 669	Rume Sudan × Catuaí	4	101	99	6	105	< 0.050	✗

For $\alpha = 0.05$ (95% confidence); $gl = 1$, and the critical value is 3.841. *CCC*: Colombian Coffee Collection; *BGII*: Germplasm Bank Two; *NHRO*; Observed resistant hypocotyls; *NHSO*: Observed Susceptible Hypocotyls; *NHRE*; Expected Resistant Hypocotyls; *NHSE*: Expected Susceptible Hypocotyls; *NTH*: Total Number of Hypocotyls; ✗: rejected ($p < 0.05$); ✓: not significantly different from expectation ($p \geq 0.05$).

Table S12. Chi-square goodness-of-fit test for theoretical segregation of resistance (15:1) to the Que2 isolate of *C. kahawae* based on the percentage of resistant hypocotyls of each F_2 population inoculated under controlled conditions.

CCC	HYbrids	NHRO	NHSO	NHRE	NHSE	NTH	*p value*	15:1
2019_3 – 781	Iapar-59 × Rume Sudan	179	223	101	301	402	< 0.050	✗
2019_3 – 782	Iapar-59 × Rume Sudan	143	51	182	12	194	< 0.050	✗
2019_3 – 783	Iapar-59 × Rume Sudan	107	22	121	8	129	< 0.050	✗
2019_3 – 784	Iapar-59 × Rume Sudan	119	86	193	12	205	< 0.050	✗
2019_3 – 785	Iapar-59 × Rume Sudan	114	29	135	8	143	< 0.050	✗
2019_3 – 786	Iapar-59 × Rume Sudan	153	56	196	13	209	< 0.050	✗
2019_3 – 788	Iapar-59 × Rume Sudan	140	62	190	12	202	< 0.050	✗
2019_3 – 791	Iapar-59 × Rume Sudan	87	130	204	13	217	< 0.050	✗
2019_3 – 792	Iapar-59 × Rume Sudan	103	100	191	12	203	< 0.050	✗
2019_3 – 795	Iapar-59 × Rume Sudan	111	85	184	12	196	< 0.050	✗
2019_3 – 796	Iapar-59 × Rume Sudan	151	52	191	12	203	< 0.050	✗
2019_3 – 793	Iapar-59 × Rume Sudan	19	82	95	6	101	< 0.050	✗
2019_3 – 794	Iapar-59 × Rume Sudan	12	143	146	9	155	0.442	✓
2019_3 – 745	Iapar-59 × ET.56	145	207	330	22	352	< 0.050	✗
2019_3 – 746	Iapar-59 × ET.56	91	132	210	13	223	< 0.050	✗
2019_3 – 751	Iapar-59 × ET.56	115	95	197	13	210	< 0.050	✗
2019_3 – 752	Iapar-59 × ET.56	74	113	176	11	187	< 0.050	✗
2019_3 – 753	Iapar-59 × ET.56	53	34	82	5	87	< 0.050	✗
2019_3 – 754	Iapar-59 × ET.56	105	123	214	14	228	< 0.050	✗
2019_3 – 755	Iapar-59 × ET.56	101	99	188	12	200	< 0.050	✗
2019_3 – 758	Iapar-59 × ET.56	121	96	204	13	217	< 0.050	✗
2019_3 – 759	Iapar-59 × ET.56	120	70	179	11	190	< 0.050	✗
BGII – 667	Rume Sudan × Catuaí	203	157	338	22	360	< 0.050	✗
BGII – 867	Rume Sudan × Catuaí	42	24	62	4	66	< 0.050	✗
BGII – 868	Rume Sudan × Catuaí	132	57	178	11	189	< 0.050	✗
BGII – 875	Rume Sudan × Catuaí	71	44	108	7	115	< 0.050	✗
BGII – 724	Rume Sudan × Catuaí	30	106	128	8	136	< 0.050	✗
BGII – 709	Rume Sudan × Catuaí	28	82	104	6	110	< 0.050	✗
BGII – 668	Rume Sudan × Catuaí	9	101	104	6	110	0.402	✓
BGII – 725	Rume Sudan × Catuaí	25	75	94	6	100	< 0.050	✗
BGII – 669	Rume Sudan × Catuaí	4	101	99	6	105	0.301	✓

For $\alpha = 0.05$ (95% confidence); gl = 1, and the critical value is 3.841. CCC: Colombian Coffee Collection; $BGII$: Germplasm Bank Two; $NHRO$; Observed resistant hypocotyls; $NHSO$: Observed Susceptible Hypocotyls; $NHRE$; Expected Resistant Hypocotyls; $NHSE$: Expected Susceptible Hypocotyls; NTH: Total Number of Hypocotyls; ✗: rejected ($p < 0.05$); ✓: not significantly different from expectation ($p \geq 0.05$).

Table S13. Statistical analysis of the results of the linear mixed models with and without considering the interactions for the populations Iapar-59 × Rume Sudan, Rume Sudan × Catuaí and Iapar-59 × ET.56.

**Population**	**Model**	**npar**	**AIC**	**BIC**	***p value***	
Iapar-59 × Rume Sudan	No interacction	4	-24.378			
	Interacction	5	-33.438	11.059	< 0.050	***
Rume Sudan × Catuaí	No interacction	4	-4.115			
	Interacction	5	-13.171	11.057	< 0.050	***
Iapar-59 × ET.56	No interacction	4	-43.002			
	Interacction	5	-45.429	4.427	< 0.050	*

*$npar$: number of parameters included in the models ($n = 4$: resistance to *C. kahawae* = CCC + isolate + experimental error, $n = 5$: resistance to *C. kahawae* = CCC + isolation + (CCC × isolate) + experimental error). Information criterion (AIC): This measure is used to compare the quality of two statistical models and select the most appropriate model. Small AIC values correspond to the best models. Information criterion (BIC): This measure is used to compare the selection of the two models and is similar to the AIC. Small BIC values correspond to the best models. The isolation of *C. kahawae* and the genotype–isolation interaction were considered random effects.

Table S14. Deduction of the parameters of the theoretical genetic model for the expression of resistance to *C. kahawae* in the segregating populations of *C. arabica* evaluated.

Population	Parameter	Ang29-Cam1-Que2	Contribution
	σ^2_F	0.092	100.0%
	σ^2_G	0.039	42.3%
	σ^2_I	0.033	35.6%
	$\sigma^2_{(G\times I)}$	0.013	14.7%
Iapar-59 × Rume Sudan	σ^2_e	0.007	7.4%
	H^2	0.423	-
	H^2 LCL	0.138	-
	H^2 UCL	0.745	-
	$\sigma^2_F(0.092) = \sigma^2_G(0.039) + \sigma^2_I(0.033) + \sigma^2_{(G\times I)}(0.013) + \sigma^2_e(0.007)$		
	σ^2_F	0.095	100.0%
	σ^2_G	0.061	64.2%
	σ^2_I	0.007	7.6%
	$\sigma^2_{(G\times I)}$	0.024	25.1%
Rume Sudan × Catuaí	σ^2_e	0.003	3.1%
	H^2	0.642	-
	H^2 LCL	0.194	-
	H^2 UCL	0.867	-
	$\sigma^2_F(0.095) = \sigma^2_G(0.061) + \sigma^2_I(0.007) + \sigma^2_{(G\times I)}(0.024) + \sigma^2_e(0.003)$		
	σ^2_F	0.056	100.0%
	σ^2_G	0.023	41.4%
	σ^2_I	0.019	34.0%
	$\sigma^2_{(G\times I)}$	0.007	11.9%
Iapar-59 × ET.56	σ^2_e	0.007	12.6%
	H^2	0.416	-
	H^2 LCL	0.119	-
	H^2 UCL	0.730	-
	$\sigma^2_F(0.056) = \sigma^2_G(0.023) + \sigma^2_I(0.019) + \sigma^2_{(G\times I)}(0.007) + \sigma^2_e(0.007)$		

σ^2_F: varianza fenotípica; σ^2_G: genetic variance; σ^2_I: variance of the isolation; $\sigma^2_{(G\times I)}$: variance of the interaction; σ^2_e: unexplained variance; H^2: broad-sense heritability; LCL: lower limit (95% confidence); UCL: upper limit (95% confidence).

Table S15. Deviations in the mean resistance to the three isolates of *C. kahawae* namely, Ang29, Cam1 and Que2 based on the Genotype × Isolate interactions in the populations Iapar-59 × Rume Sudan, Rume Sudan × Catuaí and Iapar- 59 × ET.56.

**Hybrids**	**CCC**	**LCL (G×I)**	**Ranking (G×I)**	**UCL (G×I)**
Iapar-59 x Rume Sudan	2019_3 – 794	-0.499	-0.263	-0.027
	SL.28	-0.413	-0.249	-0.084
	Iapar-59	-0.413	-0.249	-0.084
	Caturra	-0.386	-0.228	-0.071
	2019_3 – 793	-0.426	-0.190	0.045
	2019_3 – 781	-0.214	-0.052	0.110
	2019_3 – 791	-0.210	-0.046	0.118
	2019_3 – 792	-0.194	-0.029	0.134
	2019_3 – 784	-0.179	-0.015	0.149
	2019_3 – 785	-0.052	0.111	0.276
	2019_3 – 788	-0.041	0.123	0.287
	2019_3 – 795	-0.011	0.153	0.317
	2019_3 – 782	-0.003	0.160	0.325
	2019_3 – 786	0.004	0.169	0.333
	2019_3 – 783	0.001	0.169	0.337
	Rume Sudan	0.047	0.215	0.382
	2019_3 – 796	0.058	0.222	0.386
Rume Sudan x Catuaí	SL.28	-0.447	-0.261	-0.076
	Caturra	-0.432	-0.249	-0.067
	BGII – 669	-0.509	-0.232	0.043
	Catuaí	-0.413	-0.227	-0.042
	BGII – 668	-0.479	-0.202	0.074
	BGII – 724	-0.383	-0.106	0.170
	BGII – 725	-0.362	-0.085	0.191
	BGII – 709	-0.359	-0.082	0.194
	BGII – 667	-0.071	0.113	0.298
	BGII – 875	-0.089	0.126	0.341
	BGII – 779	-0.111	0.165	0.441
	BGII – 867	-0.094	0.182	0.459
	Rume Sudan	0.015	0.202	0.388
	BGII – 868	0.066	0.282	0.498
	BGII – 799	0.100	0.377	0.654
Iapar-59 x ET.56	Iapar-59	-0.356	-0.222	-0.088
	SL.28	-0.356	-0.222	-0.088
	Caturra	-0.324	-0.200	-0.075
	2019_3 – 746	-0.168	-0.034	0.099
	2019_3 – 745	-0.139	-0.014	0.110
	2019_3 – 754	-0.128	0.005	0.139
	2019_3 – 752	-0.126	0.007	0.140
	2019_3 – 755	-0.119	0.014	0.147
	2019_3 – 758	-0.049	0.084	0.217
	2019_3 – 751	-0.038	0.094	0.228
	2019_3 – 753	0.000	0.138	0.276
	2019_3 – 759	0.030	0.163	0.297
	ET.56	0.029	0.186	0.342

CCC: Colombian Coffee Collection; (G×I): Genotype × Isolate; *LCL*: lower limit (95% confidence); *UCL*: upper limit (95% confidence).
